# Synthesis and Evaluation of Zwitterionic Surfactants Bearing Benzene Ring in the Hydrophobic Tail

**DOI:** 10.3390/ma13081858

**Published:** 2020-04-15

**Authors:** Syed Muhammad Shakil Hussain, Ahmad Mahboob, Muhammad Shahzad Kamal

**Affiliations:** Center for integrative Petroleum Research, King Fahd University of Petroleum & Minerals, Dhahran 31261, Saudi Arabia; smshakil@kfupm.edu.sa (S.M.S.H.); ahmad.mahboob@kfupm.edu.sa (A.M.)

**Keywords:** poly(ethylene oxide), zwitterionic, thermal properties, surface properties, oilfield

## Abstract

Surfactant tolerance in the presence of mono and divalent reservoir ions, as well as the solubility of surfactant in high salinity and low salinity brine, are the two major requirements for any surfactant that is subjected to oilfield application. Herein, six poly(ethylene oxide) zwitterionic surfactants having different ionic headgroups and hydrophobic tail were synthesized for oilfield applications. They were characterized by various instrumental techniques (Fourier-transform infrared spectroscopy (FTIR), matrix-assisted laser desorption/ionization time-of-flight mass spectrometry (MALDI-ToF-MS), Nuclear Magnetic Resonance (NMR)) and the combination of these techniques allowed for us to deduce the structure. All of the surfactants revealed prominent solubility in high salinity and low salinity brine due to the presence of ethoxy units between the aromatic ring and amide group. The surfactant samples were oven aged for 90 days at reservoir temperature and a clear solution implies their excellent aqueous stability. Rendering to thermal gravimetric results, decomposition of surfactants was found to occur around 300 °C, which is higher than the reservoir temperature (≥90 °C). It was observed that the hydrophilic headgroup has no significant impact on the critical micelle concentration and other surface properties. However, the hydrophobic tail bearing benzene ring significantly alters the critical micelle concentration and other surface properties.

## 1. Introduction

Surfactants (surface active agents) are extensively applied in various oilfield applications, such as drilling fluid, well stimulation, filter cake removal, swelling inhibitor, corrosion prevention, fracturing, and enhanced oil recovery [1,2,3,4,5,6,7,8]. Surfactants tend to decrease oil/water interfacial tension (IFT) and alter the rock wettability [9,10,11,12]. Commercial petroleum-based surfactants undergo precipitation because of high temperature (≥90 °C) and high salinity brines (120,000–220,000 ppm) in carbonate reservoirs. The harsh environment of a reservoir causes surfactant decomposition and it reduces their ability in lowering IFT and in altering rocks wettability.

Surfactant adsorption on the reservoir rocks is a big challenge that requires broad studies before the application of surfactant in a specific reservoir. For instance, negatively charged anionic surfactants are avoided in carbonate reservoirs and the positively charged cationic surfactants are avoided in sandstone because of adsorption issues. Zwitterionic surfactants belong to the class of surfactants that is composed of both positive and negative charges, and these charges neutralize each other under the normal environment. In comparison with zwitterionic surfactants, anionic and cationic surfactants exhibit lower heat stability and lower salt tolerance due to headgroup interactions, which makes zwitterionic surfactants more suitable for oilfield applications [13]. 

Kumar et al. reported the adsorption, imbibition, and wettability alteration studies of zwitterionic surfactant on carbonate and sandstone rocks. They concluded that the zwitterionic surfactants can be effectively applied in both carbonate and sandstone reservoirs [14]. Zwitterionic surfactants found to be compatible with other classes of surfactants and they exhibit good stability in hard water as well as in acids and bases. Recently, Kurnia et al. studied the zwitterionic-anionic surfactant hybrid system in alkali-free flooding and achieved ultralow IFT, along with the displacement of 63–75% of crude oil in coreflooding tests [1]. The zwitterionic betaine displays great potential as a fracturing fluid. For example, EDAS {3-(*N*-erucamidopropyl-*N,N*-dimethyl ammonium) propane sulfonate} exhibit viscoelastic properties and meet the requirements of hydraulic fracturing operation [15]. Similarly, Zhang et al. synthesized gemini zwitterionic surfactant containing the sulfonic head group. They emphasized that the gemini zwitterionic surfactant is a better choice when compared to positively charged gemini because of surfactants due to ultra-low sensitivity towards salts and it can be applied as a seawater-based clean fracturing fluid [16]. Zwitterionic surfactant with viscoelastic properties is successfully applied in the acid diversion for well stimulation. Al-Sadat et al. developed an acid diversion system that was based on carboxybetaine zwitterionic surfactant. The research revealed that the 7.5 wt.% surfactant showed the highest elastic strength and the divalent ions, including CaCl_2_ and MgCl_2_, enhance the elasticity more than monovalent ions, such as KCl, NaCl, and NH_4_Cl [13]. The effect of hydrophilic headgroup on surface/interface and thermal properties is rare in the literature. Wang and et al. examined the influence of different hydrophilic headgroups onto the rheological properties, surface activities, as well as Krafft temperature (*T*_K_) [17]. Likewise, Dong et al. observed that the surfactant containing the carboxybetiane headgroup exhibited slightly higher CMC values in comparison with the surfactant containing hydroxy sulfobetaine headgroup at any given pH due to the more hydrophilic nature of carboxylate head (-CO_2_) when compared to sulfonate head (-SO_3_) [18]. 

When considering the above properties, the development of new zwitterionic surfactants is highly desirable. The incorporation of a suitable hydrophobic tail and the hydrophilic head is the prerequisite for acquiring the essential surface/interface and thermal properties. In this work, six betaine based zwitterionic surfactants with various hydrophobic tail containing benzene ring and hydrophilic headgroups, namely: 4-*tert*-butylphenyl ethoxylate amidopropyl carboxybetaine (TEAC), 4-*tert*-butylphenyl ethoxylate amidopropyl sulfobetaine (TEAS), 4-*tert*-butylphenyl ethoxylate hydroxy sulfobetaine (TEAH), 4-nonylphenyl ethoxylate amidopropyl carboxybetaine (NEAC), 4-nonylphenyl ethoxylate amidopropyl sulfobetaine (NEAS), and 4-nonylphenyl ethoxylate amidopropyl hydroxy sulfobetaine (NEAH) were prepared. The chemical structures were deduced with the aid of matrix-assisted laser desorption/ionization time-of-flight mass spectrometry (MALDI-ToF-MS), Fourier-transform infrared spectroscopy (FTIR), 1H and 13C Nuclear Magnetic Resonance (NMR) spectroscopy. The influence of various headgroups (carboxylate, sulfonate, and hydroxy sulfonate), as well as the hydrophobic tail (*tert*-butyl and nonyl), onto the surface/interface and thermal behavior, was identified. The salt resistance capabilities of the TEAC, TEAS, TEAH, NEAC, NEAS, and NEAH were studied for 90 days at 90 °C in seawater (SW), formation water (FW), and distilled water (DW). The surfactant structure was carefully designed to acquire certain properties. For example, the addition of a benzene ring in the hydrophobic tail leads to lower CMC values in comparison with the corresponding surfactant with alkyl hydrophobic tail [19]. The ethoxy units (-O-CH_2_-CH_2_-) were incorporated to achieve good solubility in all kinds of water through hydrogen bonding [20]. The amide functionality (-NH-C(O)-) is known to provide biodegradability, low CMC, and environmentally friendly properties [21].

## 2. Materials and Synthesis 

### 2.1. Materials

Glycolic acid ethoxylate 4-tert-butylphenyl ether (average M_n_ ~380), 3-(dimethylamino)-1-propylamine (99%), 1,3-propanesultone (98%), glycolic acid ethoxylate 4-nonylphenyl ether (average M_n_ ~600), 3-chloro-2-hydroxypropanesulfonic acid sodium salt (95%), 1,3-propanesultone (98%), sodium chloroacetate (98%), NaF (sodium fluoride, ≥99%), and Al_2_O_3_ (aluminum oxide, ≥98%) were procured from Sigma Aldrich. The dried solvents were used for the synthesis and purification of TEAC, TEAS, TEAH, NEAC, NEAS, and NEAH. High salinity brines (SW and FW) were prepared by using salts, such as NaHCO_3_ (sodium bicarbonate), CaCl_2_ (calcium chloride), NaCl (sodium chloride), MgSO_4_ (magnesium sulfate), and Na_2_SO_4_ (sodium sulfate).

### 2.2. Determination of Structure

NMR technique was conducted using a Bruker instrument (500 MHz) by using tetramethylsilane (TMS) as an internal standard. The NMR (proton and carbon-13) was done in deuterated solvents and the data were obtained in ppm. FTIR technique was conducted using the 16F model of the Perkin-Elmer instrument and FTIR data ere acquired in cm^−1^. The MALDI-ToF-MS analysis was acquired from the Bruker SolariX XR instrument (Billerica, MA, USA).

### 2.3. Solubility and Salt Tolerance Tests

15 wt.% solutions of TEAC, TEAS, TEAH, NEAC, NEAS, and NEAH were prepared in FW, DW, and SW and preserved in the oven at 90 °C for 90 days.

### 2.4. Thermal Gravimetric Analysis (TGA)

The TGA was performed with a TA instrument (SDT Q600 machine, New Castle, DE, USA) at constant warming (20 °C/min) and the range of warming was 30–500 °C in an inert atmosphere using nitrogen (100 mL/min.).

### 2.5. Surface Tension 

Surface tension measurements of surfactants (TEAC, TEAS, TEAH, NEAC, NEAS, and NEAH) were conducted on a force tensiometer (Kruss, Hamburg, Germany) that was equipped with an automatic diluter. The “Du Noüy ring” method was used for surface tension measurements in which the force required to raise the platinum ring, dipped in the liquid, out of the surface is measured and it is related to the liquid’s surface tension. Experiments were performed at 30 ± 0.1 °C The Du Noüy ring was washed using deionized water and then burnt to remove contamination and adsorbed surfactant. A test of surface tension measurement of deionized water was run before each sample run to ensure proper cleaning.

### 2.6. Synthesis

#### 2.6.1. Synthesis of Intermediate 5

**Scheme 1:**
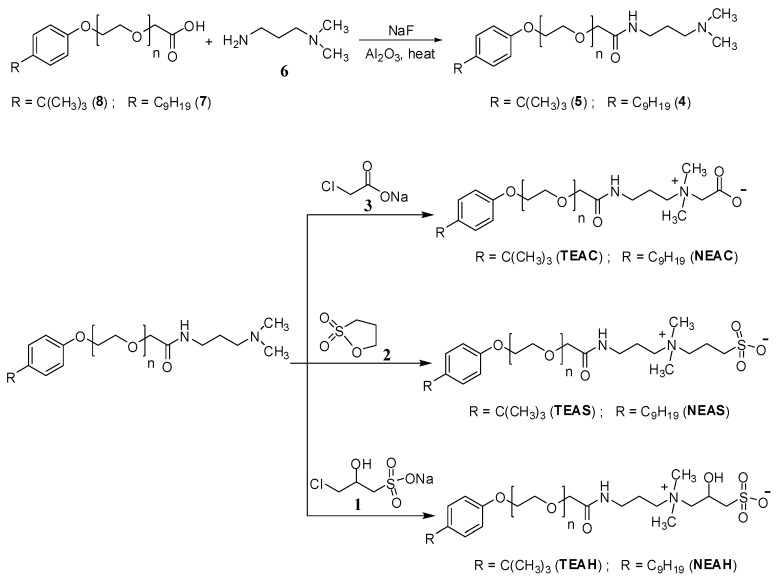
Synthesis of zwitterionic surfactants having various head and tail groups.

**Figure 1:**
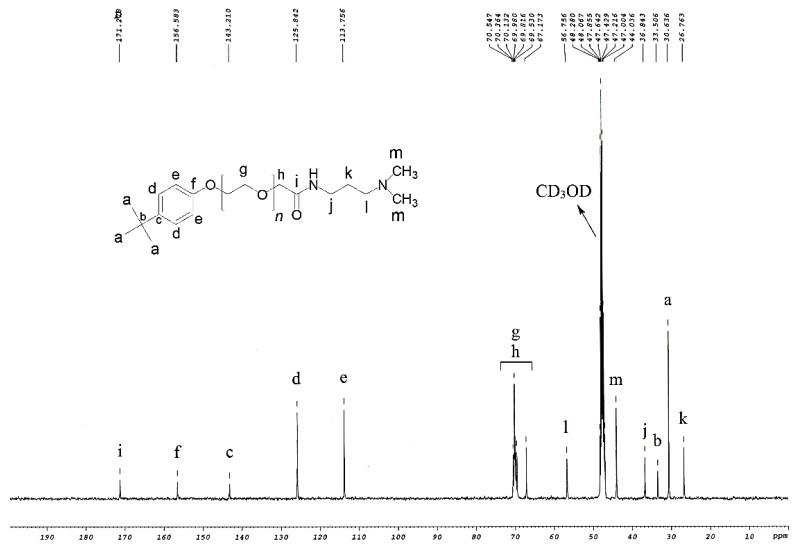
Carbon-13 Nuclear Magnetic Resonance (NMR) of the intermediate compound (5).

Intermediates 5 (Figure 1) was synthesized through the amidation of glycolic acid ethoxylate 4-tert-butylphenyl ether 8 (50 g, 131.58 mmol) with 3-(dimethylamino)-1-propylamine 6 (26.89 g, 263.16 mmol) using NaF (0.55 g, 13.16 mmol) in 250 mL 2-necked RB flask for 8 h at 160 °C in an inert atmosphere using argon (Scheme 1). The produced water of the reaction was collected using aluminum oxide. After 8 h, more 3-(dimethylamino)-1-propylamine (20.17 g, 197.37 mmol) was added and continue stirring up to 6 h. After that, excessive 3-(dimethylamino)-1-propylamine was evaporated by reduced pressure and solid NaF was separated by filtration. The resulting product was dried to obtain intermediate 5 in dark yellowish color [22,23].

The intermediate 4 was also synthesized while using the same procedure starting with glycolic acid ethoxylate 4-nonylphenyl ether 7. 

#### 2.6.2. Synthesis of 4-Tert-Butylphenyl Ethoxylate Amidopropyl Carboxybetaine (TEAC)

Intermediate 5 (15 g, 32.33 mmol) was refluxed with sodium chloroacetate (4.71 g, 40.41 mmol) in 250 mL 2-necked RB flask while using 120 mL ethanol: water (5:1) as a solvent for 12 h at 85 °C. After that, the solvent was removed by evaporation and the column chromatography was performed through an ethanol-based mobile phase to achieve TEAC in gel-like material [24]. 

The NEAC was also prepared by following the above procedure while using intermediate 4.

#### 2.6.3. Synthesis of 4-Tert-Butylphenyl Ethoxylate Amidopropyl Sulfobetaine (TEAS)

Intermediate 5 (15 g, 32.33 mmol) was refluxed with 1,3-propanesultone (5.92 g, 48.49 mmol) in 250 mL 2-necked RB flask using EtOAc (150 mL) as a solvent for 12 h at 80 °C. After that, the solvent was removed by evaporation and the column chromatography was performed through an ethanol-based mobile phase in order to achieve TEAS in gel-like material [22]. 

The NEAS was also prepared by following the above procedure using intermediate 4.

#### 2.6.4. Synthesis of 4-Tert-Butylphenyl Ethoxylate Amidopropyl Hydroxy Sulfobetaine (TEAH)

Intermediate 5 (15 g, 32.33 mmol) was refluxed with 3-chloro-2-hydroxypropanesulfonic acid sodium salt (6.99 g, 35.56 mmol) and sodium carbonate (3.43 g, 32.33 mmol) in 250 mL 2-necked RB flask while using 100 mL of 70% aqueous ethanol as a solvent for 12 h at 85 °C. After that, the solvent was removed by evaporation and the column chromatography was performed through an ethanol-based mobile phase to achieve TEAH in gel-like material [25].

The NEAH was also prepared by following the above procedure using intermediate 4.

## 3. Results and Discussion

Scheme 1 represents the synthesis of zwitterionic surfactants (TEAC, TEAS, TEAH, NEAC, NEAS, NEAH). Glycolic acid ethoxylate 4-*tert*-butylphenyl ether (8) was stirred with 3-(dimethylamino)-1-propylamine (6) while using NaF at 160 °C. The resulting compound 5 was separately reacted with 3-chloro-2-hydroxypropanesulfonic acid sodium salt (3), 1,3-propanesultone (2), and sodium chloroacetate (1) to achieve the related TEAH, TEAS, and TEAC, respectively.

NEAC, NEAS, and NEAH were also prepared by following a similar procedure using intermediate 4.

### 3.1. Structure Interpretation 

**Figure 2:**
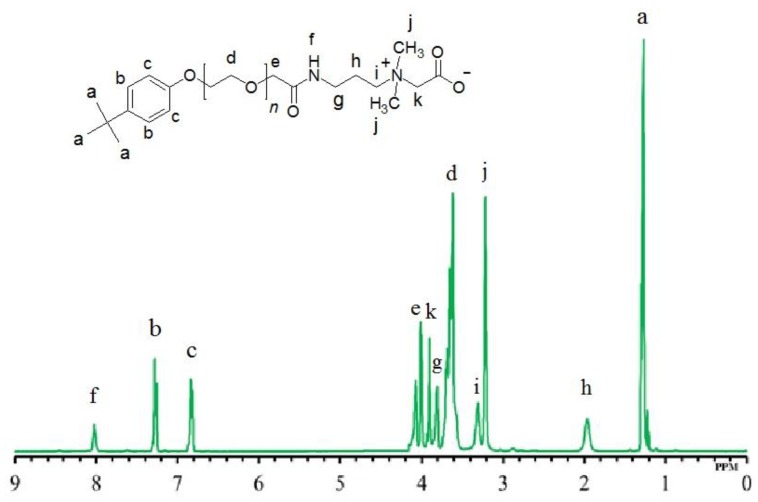
1H NMR of TEAC.

The chemical structures of all the synthesized zwitterionic surfactants (TEAC, TEAS, TEAH, NEAC, NEAS, and NEAH) and corresponding intermediates were identified using MALDI-ToF-MS, NMR, and FTIR. We described the structure identification of TEAC here as an example. Rendering the proton NMR data of TEAC (Table 1, Figure 2), the intense peak at δ 1.26 ppm was referred to the protons of three methyl units of tertiary butyl group [C(CH_3_)_3_] attached with the phenyl ring. The peak at δ 3.21 ppm could be related to two methyl units of positively charged ammonium head [N^+^(CH_3_)_2_]. The signals of the ethoxy group appeared as an overlapped peak at δ 3.60–3.69 ppm. The doublet peaks at δ 6.82 ppm were linked with the two aromatic protons of phenyl ring [2 × (CH)] at meta position with respect to the tertiary butyl group. Similarly, doublet peaks at δ 7.26 ppm could be linked with the next two aromatic protons of phenyl ring [2 × (CH)] at the ortho position with respect to the tertiary butyl group. The broad singlet peak at δ 8.02 ppm could be assigned to an amide group [NH(CO)]. 

**Figure 3:**
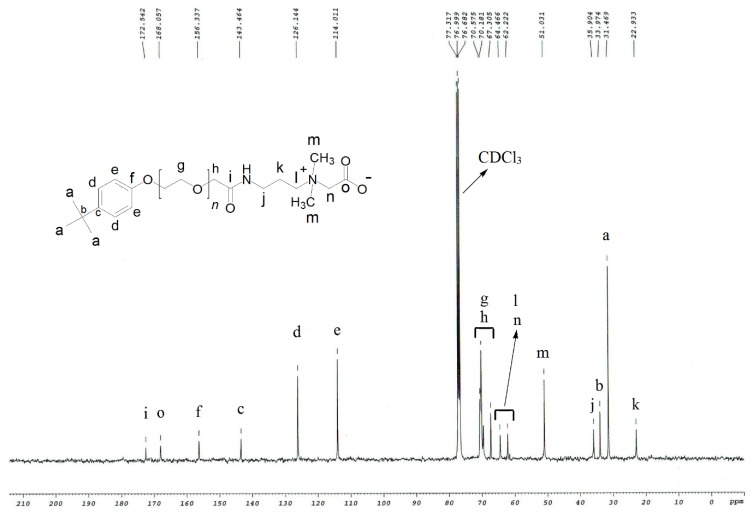
Carbon-13 NMR of TEAC.

Rendering to carbon-13 NMR data of TEAC (Table 2, Figure 3), the strong peak at δ 31.5 ppm referred to carbons of three methyl units of tertiary butyl group [C(CH_3_)_3_] attached to the phenyl ring. The peak at δ 34.0 ppm was assigned to a quaternary carbon of tertiary butyl [C(CH_3_)_3_] connected with the phenyl ring. The peak at δ 51.0 ppm was related to two methyl units of positively charged ammonium head [-N^+^(CH_3_)_2_]. The signals that were observed at δ 62.2 ppm and δ 64.5 ppm were referred to two methylene units of positively charged ammonium head [(CH_2_)_2_-N^+^(CH_3_)_2_]. The overlapped signals of methylene units of the ethoxy group were detected at δ 67.3–70.6 ppm. The signals at δ 114.0 ppm referred to two methine carbons of phenyl ring [2 × (CH)] at meta position with respect to the tertiary butyl group. Similarly, the peak at δ 126.1 ppm was linked with the next two methine carbons of phenyl ring [2 × (CH)] at the ortho position with respect to the tertiary butyl group. The peak at δ 143.5 ppm was assigned to a quaternary carbon of phenyl ring [C-C-(CH_3_)_3_] that was directly bonded with the tertiary butyl group. Likewise, the peak at δ 156.3 ppm was associated with the quaternary carbon of phenyl ring [C-O-CH_2_-CH_2_-] that was directly bonded with the ethoxy group. Two peaks detected at δ 168.1 ppm and δ 172.5 ppm were linked with the carbonyl carbon of acetate [CH_2_COO] and carbonyl carbon of amide [CH_2_CONH], respectively. 

**Figure 4:**
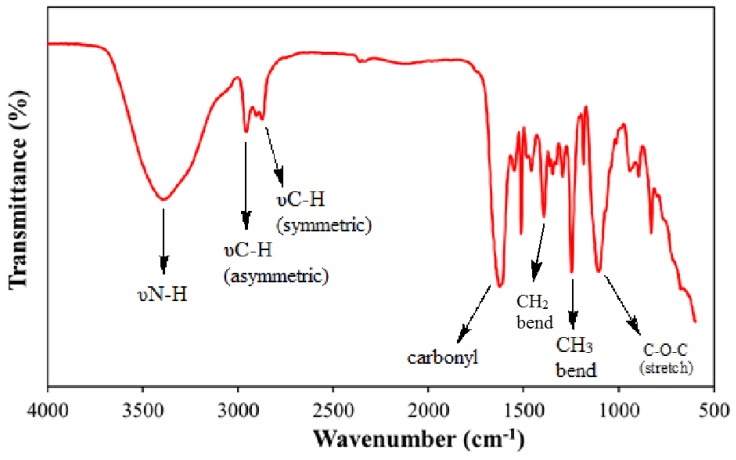
Fourier-transform infrared spectroscopy (FTIR) of TEAC.

Rendering to the FTIR spectra of TEAC (Table 3, Figure 4), strong adsorption at 3388 cm^−1^ related to NH stretch. Two adsorption signals at 2959 cm^−1^ and 2872 cm^−1^ are allocated to the CH_3_ and CH_2_ stretching regions, respectively. The strong signals at 1625 cm^−1^ were assigned to the carbonyl stretch. The adsorption signals at 1460 cm^−1^ may refer to CH_2_ bend. The ethoxy groups were detected by the adsorption signals at 1106 cm^−1^ [26]. 

**Figure 5:**
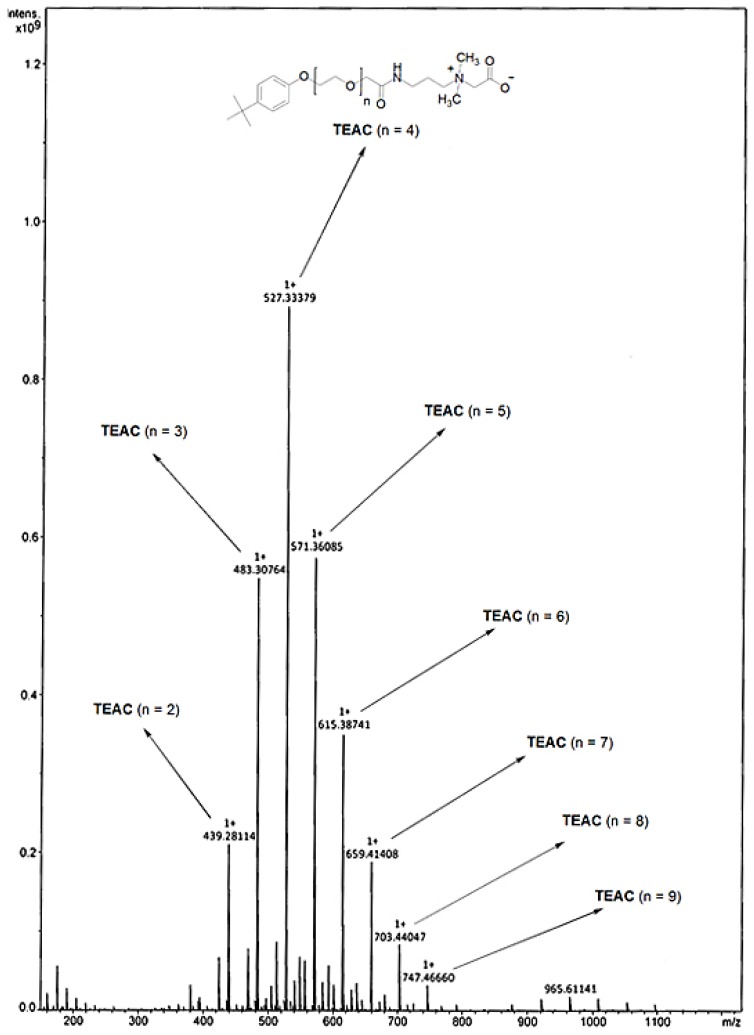
Matrix-assisted laser desorption/ionization time-of-flight (MALDI-ToF) mass spectra of TEAC.

Generally, the NMR and FTIR techniques are sufficient for identifying the chemical structure of the surfactant. However, for high molecular weight surfactants containing the distribution of ethoxy units, the combination of an additional method, such as MALDI-ToF-MS, is suitable for achieving insight into the chemical structure of surfactant. According to MALDI-ToF-MS of TEAC (Figure 5, Table 4), the base peak of *m*/*z* 527.33 can be assigned to an exact molecular mass of TEAC containing 4 ethoxy units (n = 4). The following peaks before and after the major peak are the distribution of ethoxy units with the mass difference of *m*/*z* 44, which is a molecular mass of one ethoxy unit. For example, the peak of *m*/*z* 439.28 is assigned to a molecular mass of TEAC containing two ethoxy units (n = 2) and the peak of *m*/*z* 483.31 is referred to as a molecular mass of TEAC having three ethoxy units (n = 3). Similarly, the signals of *m*/*z* 571.36 are assigned to a molecular mass of TEAC with five ethoxy units (n = 5) and the peak of *m*/*z* 615.39 is referred to as a molecular mass of TEAC having six ethoxy units (n = 6). 

Overall, the NMR, FTIR, and MALDI-ToF-MS data appeared to be in agreement with the suggested structure of TEAC. The detailed structural characterization data is available in supporting information (Figures S1–S20).

### 3.2. Solubility and Salt Tolerance

**Figure 6:**
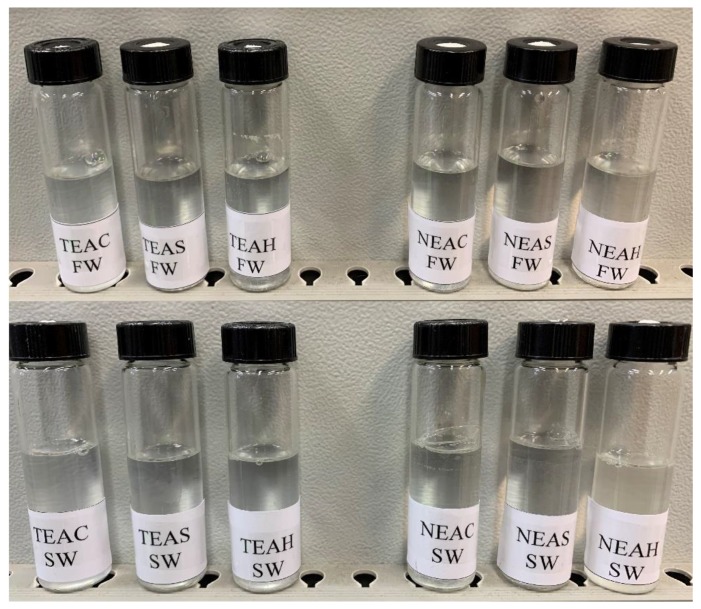
Snapshot of oven aged samples of zwitterionic surfactants at 90 °C for 90 days in seawater (SW) and reservoir brine (FW).

The tolerance of surfactant in mono and divalent reservoir ions, and the solubility of surfactant in a normal and high saline environment, are the two major requirements for any surfactant subjected to oilfield application. The synthesized zwitterionic surfactants (TEAC, TEAS, TEAH, NEAC, NEAS, and NEAH) showed good solubility in low salinity and high salinity brine due to the presence of ethoxy units between the aromatic ring and positively charged ammonium head [27]. The enhancement of surfactant solubility because of the presence of ethoxy units might be attributed to the formation of the hydrogen bond between partially negative oxygen of ethoxy units and partially positive hydrogen of water molecules [28]. The aqueous stability and salt tolerance tests were conducted by solubilizing TEAC, TEAS, TEAH, NEAC, NEAS, and NEAH samples in DW, FW, as well as SW and oven aged for 90 days at 90 °C. Table 5 presents the salt concentration in simulated FW and SW. Heating the surfactant solution can enhance solubility, but it has a negative effect on aqueous stability. All of the synthesized zwitterionic surfactants (TEAC, TEAS, TEAH, NEAC, NEAS, NEAH) showed prominent aqueous stability in all kinds of water. A transparent solution after 90 days of aging samples at 90 °C revealed that TEAC, TEAS, TEAH, NEAC, NEAS, and NEAH are stable in normal and high salinity brine (Figure 6). 

### 3.3. Thermal Gravimetric Analysis (TGA)

**Figure 7:**
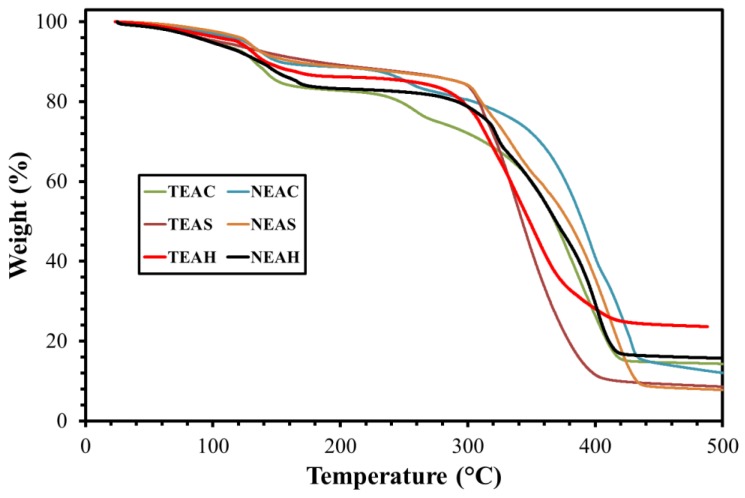
Thermal Gravimetric Analysis (TGA) curves of zwitterionic surfactants.

During the oilfield application, the solution of a surfactant is injected into the reservoir and remains there in a non-ambient environment for several days. Therefore, thermal studies of the synthesized surfactant are important for identifying the efficiency of the surfactant in a harsh environment. The thermal degradation behavior for TEAC, TEAS, TEAH, NEAS, NEAC, and NEAH was performed by TGA. Rendering to TGA isotherm (Figure 7), the initial 10–15% weight loss for all surfactants was due to the moisture and impurities. A big slope was detected at 330 °C (TEAC), 302 °C (TEAS), 290 °C (TEAH), 345 °C (NEAC), 304 °C (NEAS), and 313 °C (NEAH), indicating the effect of heat on the decomposition of structure of surfactants. All of the synthesized surfactants exhibited a similar degradation pattern. In general, it is concluded that the synthesized surfactants revealed higher degradation temperature than the actual reservoir temperature (90 °C) and the nature of hydrophilic head and the hydrophobic tail has a slight influence on heat stabilities of the surfactant.

### 3.4. Surface Tension

**Figure 8:**
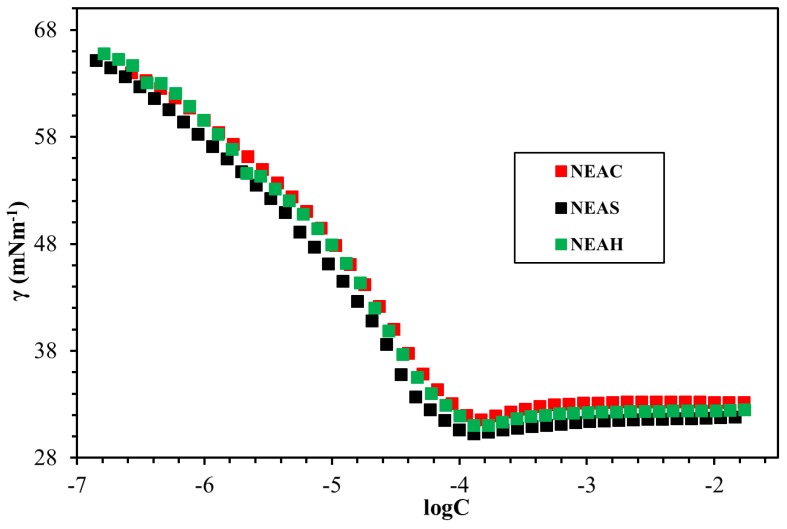
The surface tension-logC plots of NEAC, NEAS, and NEAH in distilled water (DW) at 30 °C.

**Figure 9:**
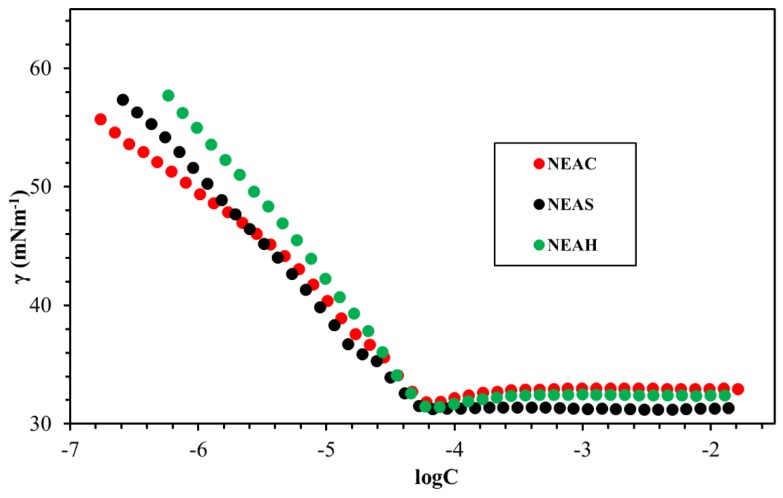
The surface tension-logC plots of NEAC, NEAS, and NEAH in 1M NaCl at 30 °C.

**Figure 10:**
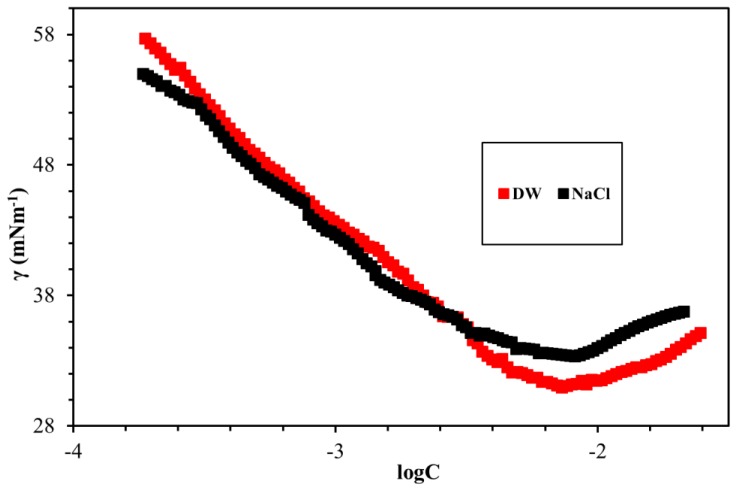
Comparison of surface tension of TEAC DW and in 1M NaCl at 30 °C.

**Figure 11:**
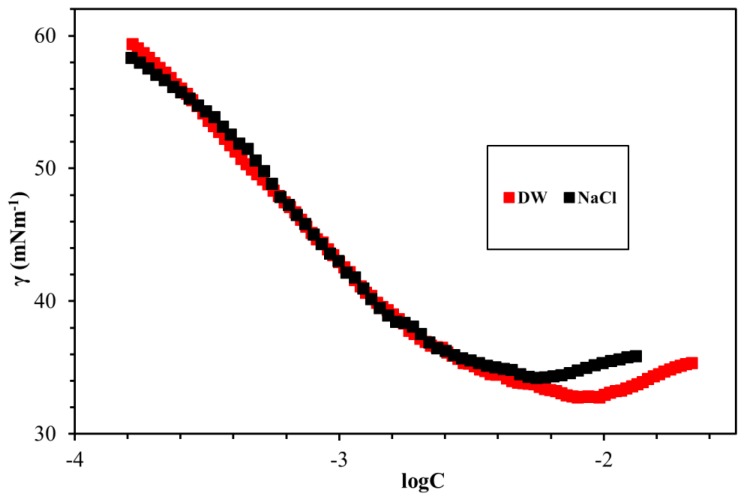
Comparison of surface tension of TEAH in DW and in 1M NaCl at 30 °C.

**Figure 12:**
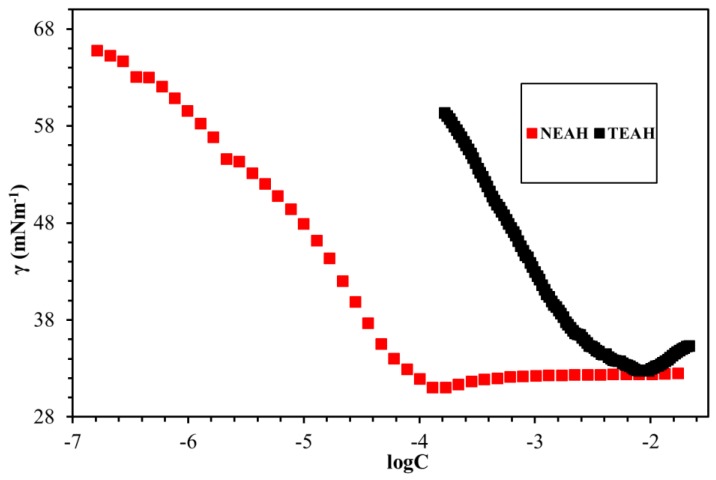
The effect of the tail on the of surface tension in DW at 30 °C.

The fundamental surface/interface properties of the synthesized surfactants were measured while using the Du Noüy ring method, as outlined in Figure 8, Figure 9, Figure 10, Figure 11 and Figure 12 and Table 6. The surface tension of all surfactants decreased with increasing surfactant concentration until it reached a critical micelle concentration. Figure 8 displayed the surface tension of NEAC, NEAS, and NEAH in deionized water at 30 °C and no significant difference in CMC was observed (Table 6). Figure 9 exhibited the surface tension of NEAC, NEAS, and NEAH in 1M NaCl solution. In the comparison of surface properties in deionized water and in 1M NaCl solution, it was observed that the CMC of surfactant solution in 1M NaCl is significantly lower than the CMC of surfactant solution in deionized water. This behavior was consistent for all three types of headgroups. The added salts promote the adsorption of polar groups at the interface of air-water because of reduced hydration. In addition, the presence of salts also facilitates more closed packing at the interface, which resulted in lowering CMC. Figure 10 and Figure 11 highlight the effect of salinity on surface properties of TEAC and TEAH, respectively. For both surfactants, the CMC decreased with an increase in salinity; however, surface tension at CMC (γ_cmc_) increased with an increase in salinity. The data that are given in Table 6 indicate the effect of the hydrophobic tails and hydrophilic head on the CMC. The change in the head group has an insignificant effect on the CMC. However, changing the hydrophobic tail significantly alters the CMC. For example, the CMC of NEAH and NEAC are like each other with a negligible difference. Similarly, the CMC of TEAH is slightly lower than that of TEAC due to different chemical structures. A similar slight difference between CMC has been reported earlier for different head groups by Dong et al. [18]. Figure 12 describes the influence of the hydrophobic tail on the surface properties of the surfactants containing an identical headgroup. The CMC of NEAH (0.1308 mM) containing a C9 tail was significantly lower than the CMC of the TEAH (3.828) having a C4 tail. Similarly, the surface tension of NEAH was found to be much lower when compared to the surface tension of TEAH [29].

The tendency of these synthesized surfactants to be adsorbed at the air-water interface was determined using maximum surface excess (г*_max_*) and minimum area per molecule (*A_min_*). The parameter *A_min_* represents the area that is occupied by a surfactant molecule at the air/solution interface. A higher *A_min_* value indicates that a single molecule will occupy more space at the interface and the number of molecules will be lesser at the interface and vice versa. These properties were determined using Equations (1) and (2):

(1) $${г}_{max}=-\frac{1}{\;nRT}{\left[\frac{d\gamma }{\mathrm{dlnC}}\right]}_{T}$$

(2) $$A_{min}=\frac{10^{18}}{N_{A}{г}_{max}}$$

Here, dγ/dlnC represents the gradient below cmc, *R* is gas constant, *T* is temperature, and *N_A_* is Avogadro number [30]. The added salts reduced the maximum surface excess and increase the *A_min_* for all surfactants (TEAC, TEAS, TEAH, NEAC, NEAS, and NEAH). Surfactants with a longer tail (NEAC, NEAS, and NEAH) exhibited lower г*_max_* values when compared to the corresponding surfactant having a shorter tail (TEAC, TEAS, TEAH). Similarly, surfactants with a longer tail (NEAC, NEAS, and NEAH) showed high *A_min_* values as compared to the surfactants containing a shorter tail (TEAC, TEAS, TEAH).

## 4. Conclusions

We demonstrated the influence of ionic headgroup and hydrophobic tail containing a benzene ring on the thermal and the surface properties of the synthesized poly(ethylene oxide) zwitterionic surfactants. The surfactants revealed prominent solubility in low salinity and high salinity brine and the oven aged samples of surfactants for 90 days at reservoir temperature showed no phase separation and/or precipitation, which confirm their excellent aqueous stability. TGA data revealed a higher degradation temperature than the actual reservoir temperature (90 °C) and the nature of the hydrophilic head and hydrophobic tail showed little effect on the thermal properties of the surfactants. It was established that the impact of various headgroups was not very obvious on CMC and other surface properties of the surfactants. However, the variation of the hydrophobic tail significantly alters the CMC and other surface properties. For instance, the CMC values reduced considerably by increasing the length of the hydrophobic tail in both normal as well as high saline water and NEAC exhibited lowest CMC (0.0507 mmol L^−1^) in 1M NaCl. Moreover, a reduction in CMC was also observed by using high salinity brine. On the other hand, γ_cmc_ increased by increasing the hydrophobic tail length, which emphasized that the surface properties primarily depend on the hydrophobic tail rather than hydrophobic headgroup. The synthesized zwitterionic surfactants exhibited superior surface and thermal properties and could be a suitable candidate in harsh reservoir conditions.

## Figures and Tables

**Table 1:** ^1^H NMR values and peak assignment of zwitterionic surfactants.

ZwitterIonic Surfactants	^1^H NMR Values (500 MHz, δ in ppm, CDCl_3_)															
	Lipophilic Tail				Ethoxy Units	Acetic	Amide	Amido-Amine							Benzene Ring	
	3 × CH_3_	CH_3_CH_2_	CH_2_	CH_2_	2 × CH_2_	CH_2_	NH	2 × CH_3_	CH_2_	CH_2_	CH_2_	CH_2_	CH_2_	CH_2_	2 × CH	2 × CH
**TEAC**	1.27	-	-	-	3.64	4.07	8.02	3.22	1.96	-	3.30	3.32	3.61	-	6.82	7.26
**TEAS**	1.27	-	-	-	3.63	4.07	8.01	3.11	1.95	2.18	2.91	3.34	3.51	3.80	6.82	7.27
**TEAH**	1.26	-	-	-	3.64	4.06	7.98	3.20	2.04	4.67 CH	3.15	3.33	3.52	3.79	6.81	7.25
**NEAC**	-	0.80	1.25	1.50	3.63	4.10	8.01	3.23	1.98	-	3.32	3.34	3.60	-	6.83	7.19
**NEAS**	-	0.77	1.23	1.53	3.64	4.09	8.00	3.10	1.99	2.17	2.89	3.34	3.49	3.82	6.82	7.20
**NEAH**	-	0.79	1.25	1.52	3.63	4.03	8.00	3.23	2.06	4.68 CH	3.15	3.34	3.53	3.80	6.82	7.18

**Table 2:** ^13^C NMR records displaying the values of chemical shift in zwitterionic surfactants.

Zwitterionic Surfactants	^13^C NMR (CDCl_3_, δ in ppm, 125 MHz)
TEAC	22.9, 31.5, 34.0, 35.9, 51.0, 62.2, 64.5, 67.3, 69.0–71.0, 114.0, 126.1, 143.5, 156.3, 168.1, 172.5
TEAS	18.5, 22.6, 31.4, 33.9, 35.8, 50.6, 60.5, 62.6, 67.2, 69.0–71.0, 113.9, 126.0, 143.4, 156.2, 171.3
TEAH	22.9, 31.5, 34.0, 35.9, 51.9, 55.3 63.0, 67.3, 67.5, 69.0–71.0, 114.0, 126.1, 143.4, 156.4, 171.3
NEAC	14.4, 22.9, 29.1, 35.9, 51.0, 62.2, 64.5, 67.2, 69.0–71.0, 113.8, 126.8, 156.1, 167.8, 172.3
NEAS	14.1, 22.7, 24.8, 29.2, 35.9, 50.8, 60.6, 62.7, 67.3, 69.0–71.0, 113.7, 126.9, 156.3, 171.5
NEAH	14.1, 22.6, 29.1, 35.6, 51.8, 55.3, 62.9, 67.2, 67.5, 69.0–71.0, 113.8, 126.9, 156.1, 171.3

**Table 3:** FTIR data of the zwitterionic surfactants.

Zwitterionic Surfactants	FTIR Data (in cm^−1^)							
	(υ_N-H_)	υ_C-H_ Asym.	υ_C-H_ Sym.	Amide (I)	CH_2_ (bend)	CH_3_ (bend)	Ethoxy Stretch	Asym. Stretch
TEAC	3398	2959	2872	1625	1393	1248	1106	941
TEAS	3406	2954	2871	1654	1393	1245	1114	925
TEAH	3405	2953	2871	1656	1393	1246	1104	926
NEAC	3405	2957	2872	1628	1393	1248	1101	944
NEAS	3404	2956	2872	1651	1349	1290	1104	947
NEAH	3405	2944	2891	1621	1371	1275	1164	973

**Table 4:** MALDI-ToF-MS analysis of zwitterionic surfactants (TEAC, TEAS, TEAH, NEAS, NEAC, and NEAH).

Zwitterionic Surfactants	Base Peak	Proposed Structure
TEAC	527.3	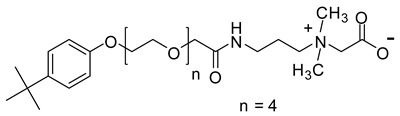
TEAS	591.3	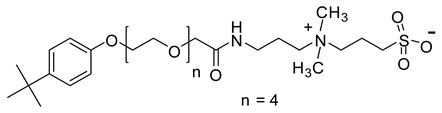
TEAH	607.3	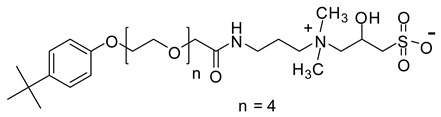
NEAC	641.4	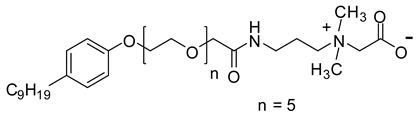
NEAS	675.4	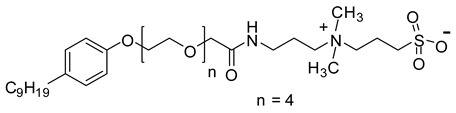
NEAH	765.5	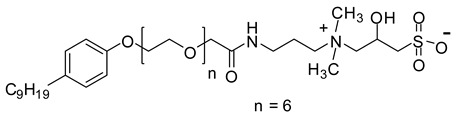

**Table 5:** The composition of seawater and formation water.

Ions	FW (g L^−1^)	SW (g L^−1^)
Na^+^	59.5	18.3
Ca^2+^	19.1	0.7
Mg^2+^	2.5	2.1
SO_4_^2−^	0.4	4.3
Cl^−^	132.1	32.2
HCO_3_^−^	0.4	0.1
Total	214	57.7

**Table 6:** Surface properties of surfactants in DW and in 1M NaCl solution at 30 °C.

Surfactant	Solvent	CMC (mmol L^−1^)	γ_cmc_ (mN m^−1^)	г*_max_* × 10^6^ (mol m^−2^)	*A_min_* (nm^2^)
NEAC	DW	0.1043	32.18	2.53	0.65
NEAS	DW	0.1045	30.53	2.35	0.70
NEAH	DW	0.1308	31.55	2.27	0.73
NEAC	NaCl	0.0507	32.34	1.95	0.89
NEAS	NaCl	0.0522	31.39	1.92	0.86
NEAH	NaCl	0.0568	31.89	2.22	0.74
TEAC	DW	6.142	30.226	3.02	0.59
TEAS	DW	6.667	28.98	2.78	0.59
TEAH	DW	3.828	31.79	3.46	0.47
TEAC	NaCl	5.792	31.43	2.35	0.70
TEAS	NaCl	6.010	29.09	2.43	0.68
TEAH	NaCl	3.163	32.79	3.63	0.45

## Supplementary Materials

The following are available online at https://www.mdpi.com/1996-1944/13/8/1858/s1, Figure S1: FT-IR spectrum of zwitterionic surfactants (TEAS), Figure S2.: ^1^H-NMR of zwitterionic surfactant (TEAS), Figure S3: ^13^C-NMR of zwitterionic surfactant (TEAS), Figure S4: MALDI-TOF-MS spectra of zwitterionic surfactant (TEAS), Figure S5: FT-IR spectrum of zwitterionic surfactants (TEAH), Figure S6: ^1^H-NMR of zwitterionic surfactant (TEAH), Figure S7: ^13^C-NMR of zwitterionic surfactant (TEAH), Figure S8: MALDI-TOF-MS spectra of zwitterionic surfactant (TEAH), Figure S9: FT-IR spectrum of zwitterionic surfactants (NEAC), Figure S10: ^1^H-NMR of zwitterionic surfactant (NEAC), Figure S11: ^13^C-NMR of zwitterionic surfactant (NEAC), Figure S12: MALDI-TOF-MS spectra of zwitterionic surfactant (NEAC), Figure S13: FT-IR spectrum of zwitterionic surfactants (NEAS), Figure S14: ^1^H-NMR of zwitterionic surfactant (NEAS), Figure S15: ^13^C-NMR of zwitterionic surfactant (NEAS), Figure S16: MALDI-TOF-MS spectra of zwitterionic surfactant (NEAS), Figure S17: FT-IR spectrum of zwitterionic surfactants (NEAH), Figure S18: ^1^H-NMR of zwitterionic surfactant (NEAH), Figure S19: ^13^C-NMR of zwitterionic surfactant (NEAH), Figure S20: MALDI-TOF-MS spectra of zwitterionic surfactant (NEAH).
