# Supplementary material for: Synthesis and Evaluation of Zwitterionic Surfactants Bearing Benzene Ring in the Hydrophobic Tail
Source: Materials (Basel). 2020 Apr 15;13(8):1858. doi: 10.3390/ma13081858 (PMC7215865; doi:10.3390/ma13081858)
Supplement: Supplementary file 1 [file materials-13-01858-s001.pdf]

materials

Supplementary Materials

Synthesis and Evaluation of Zwitterionic Surfactants Bearing Benzene Ring in the Hydrophobic Tail

Syed Muhammad Shakil Hussain, Ahmad Mahboob and Muhammad Shahzad Kamal *

Center for integrative Petroleum Research, King Fahd University of Petroleum & Minerals; smshakil@kfupm.edu.sa (S.M.S.H.); ahmad.mahboob@kfupm.edu.sa (A.M.)

* Correspondence: shahzadmalik@kfupm.edu.sa; Tel.: +966 13 8608513; Fax: +966 13 60 3989

Received: 28 March 2020; Accepted: 10 April 2020; Published: date

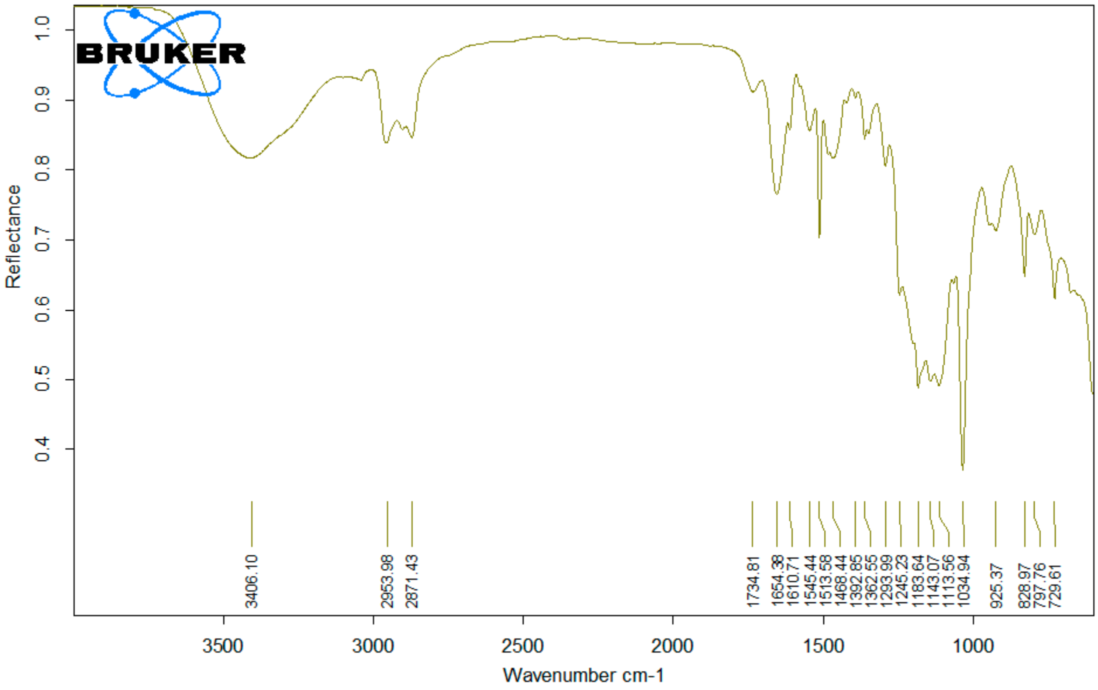

Figure S1. FT-IR spectrum of zwitterionic surfactants (TEAS).

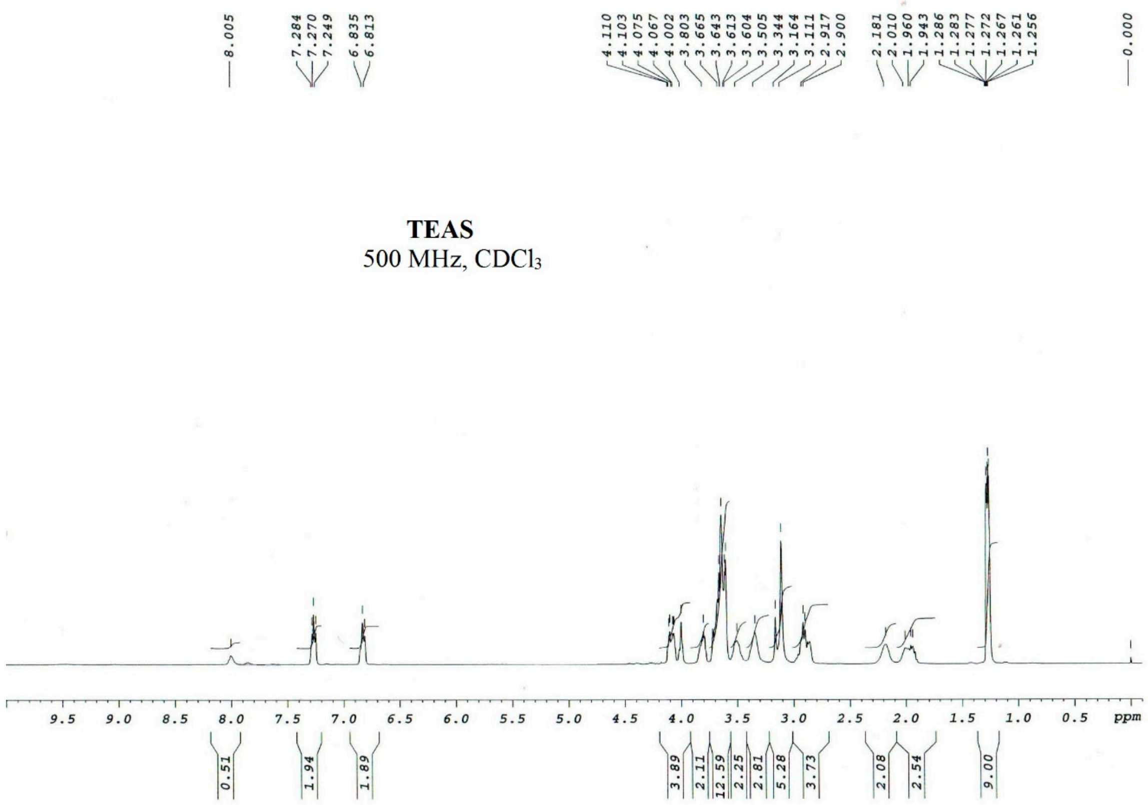

Figure S2. ^{1}H-NMR of zwitterionic surfactant (TEAS).

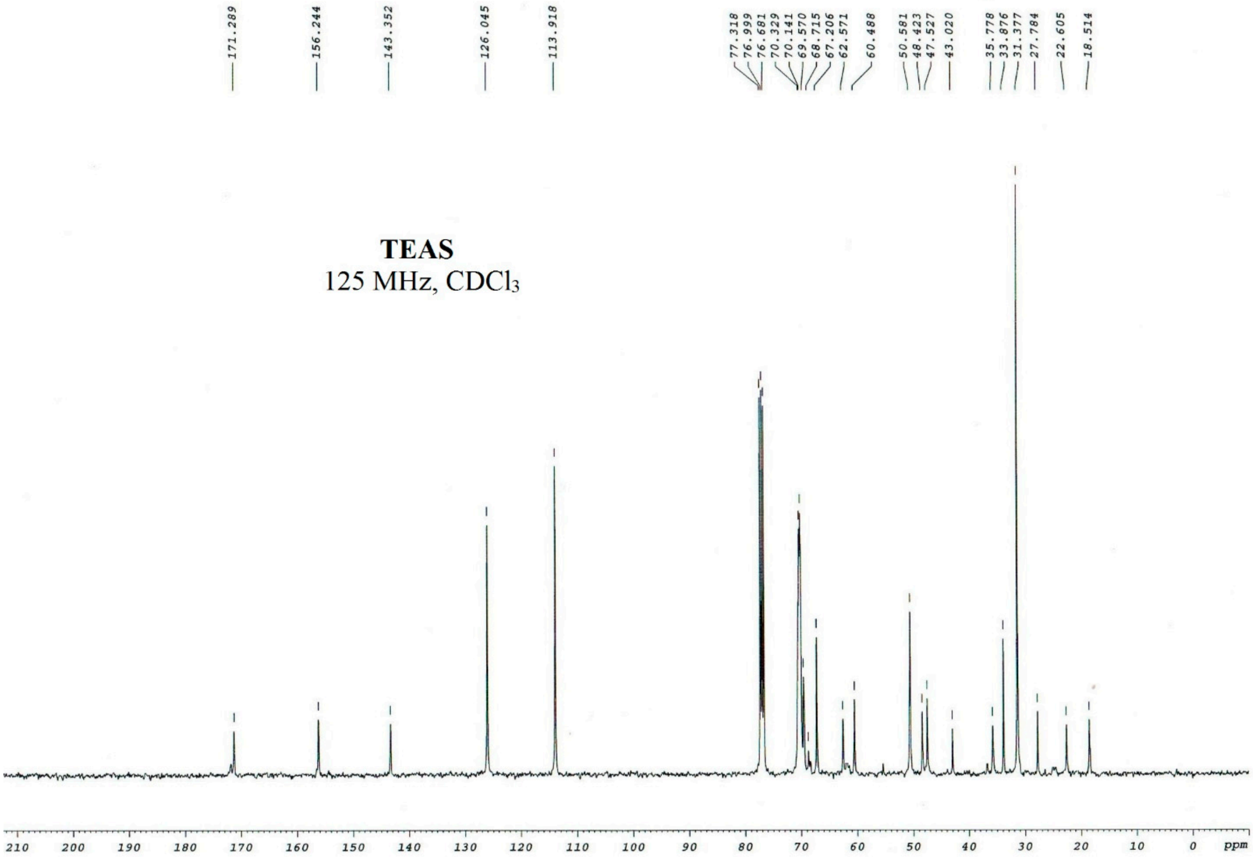

Figure S3. ^{13}C-NMR of zwitterionic surfactant (TEAS).

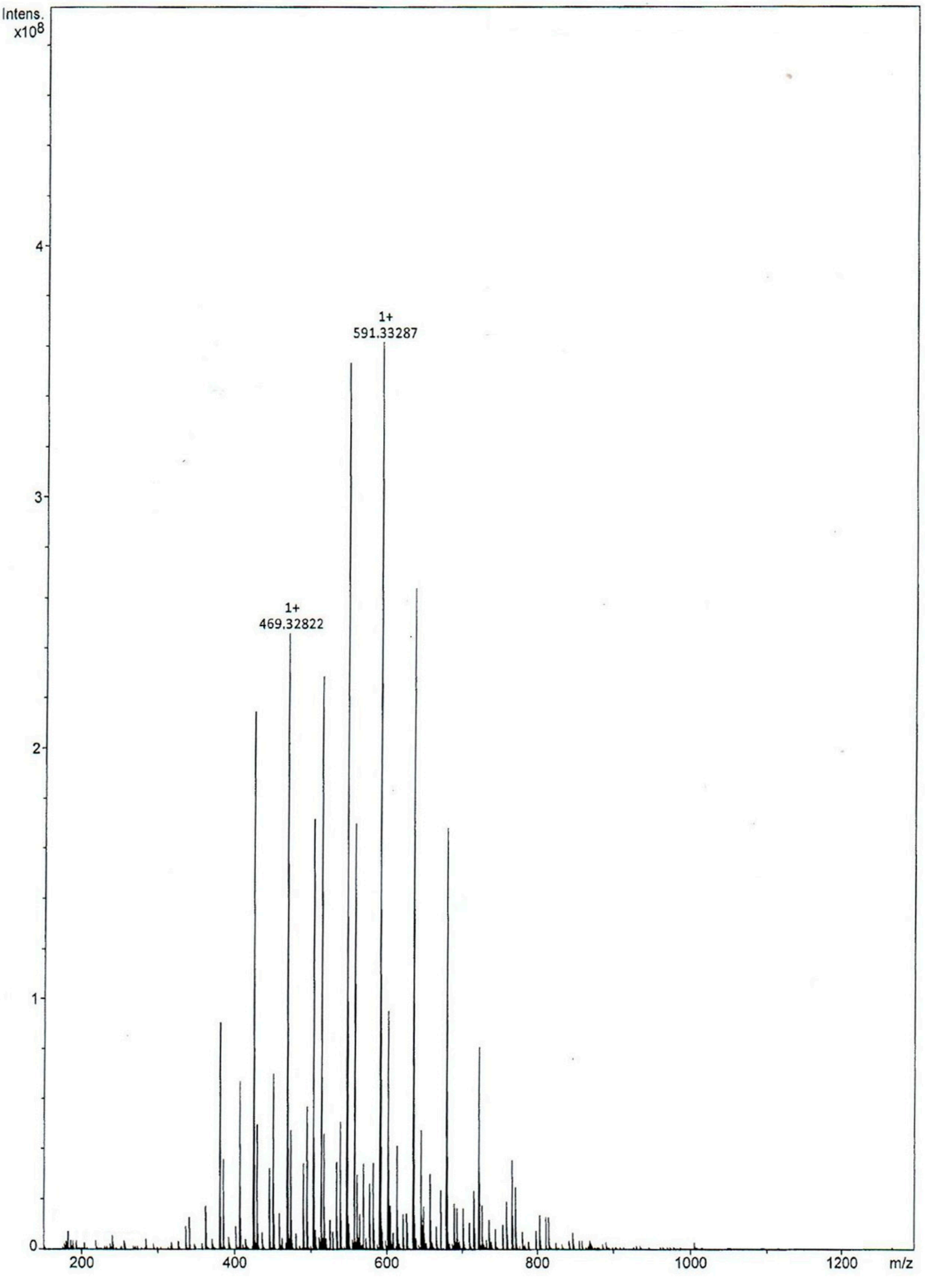

Figure S4. MALDI-TOF-MS spectra of zwitterionic surfactant (TEAS).

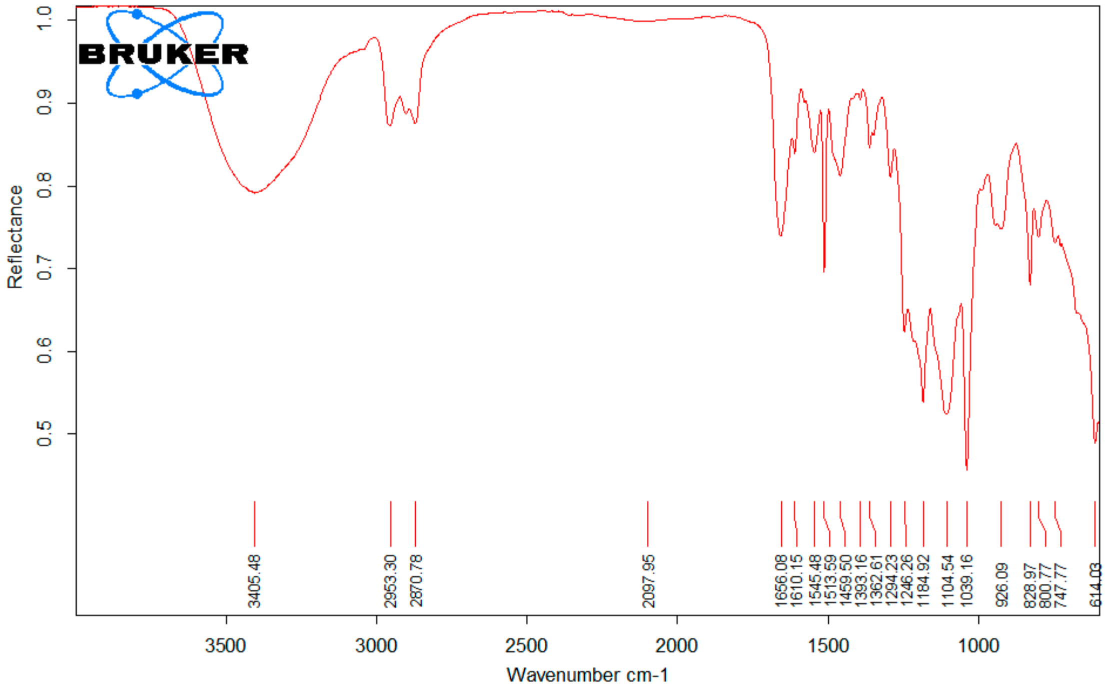

Figure S5. FT-IR spectrum of zwitterionic surfactants (TEAH).

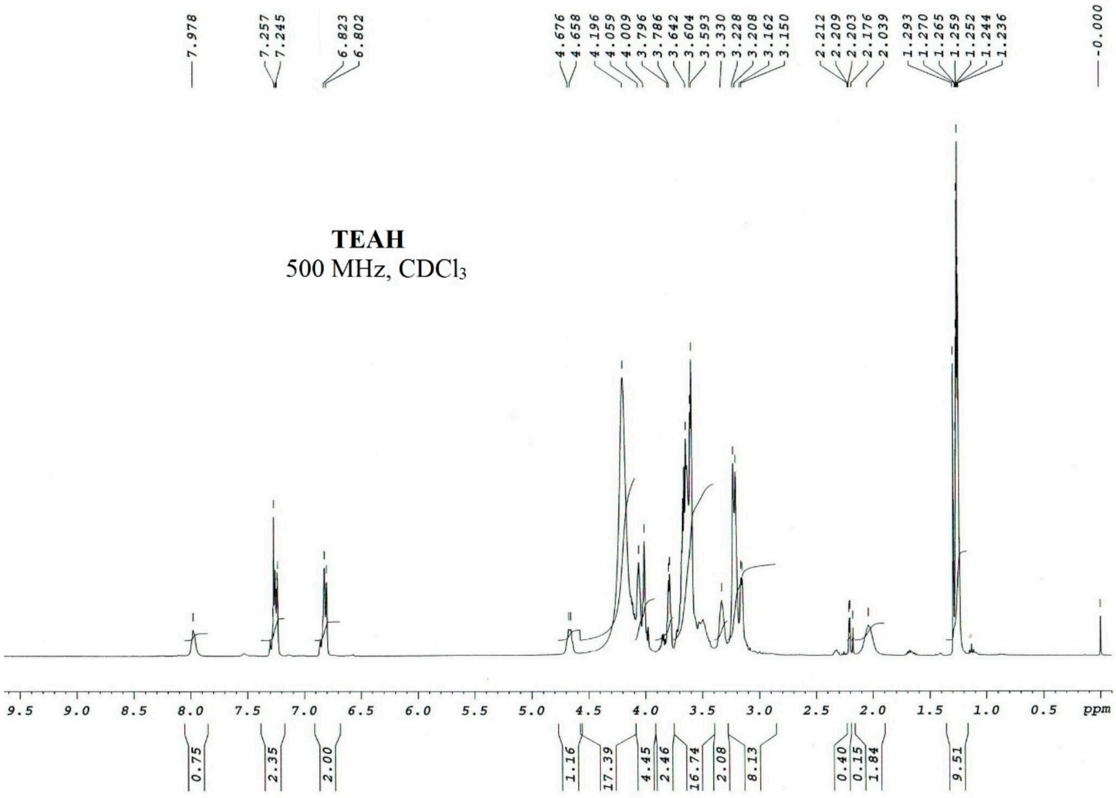

Figure S6. ^{1}H-NMR of zwitterionic surfactant (TEAH).

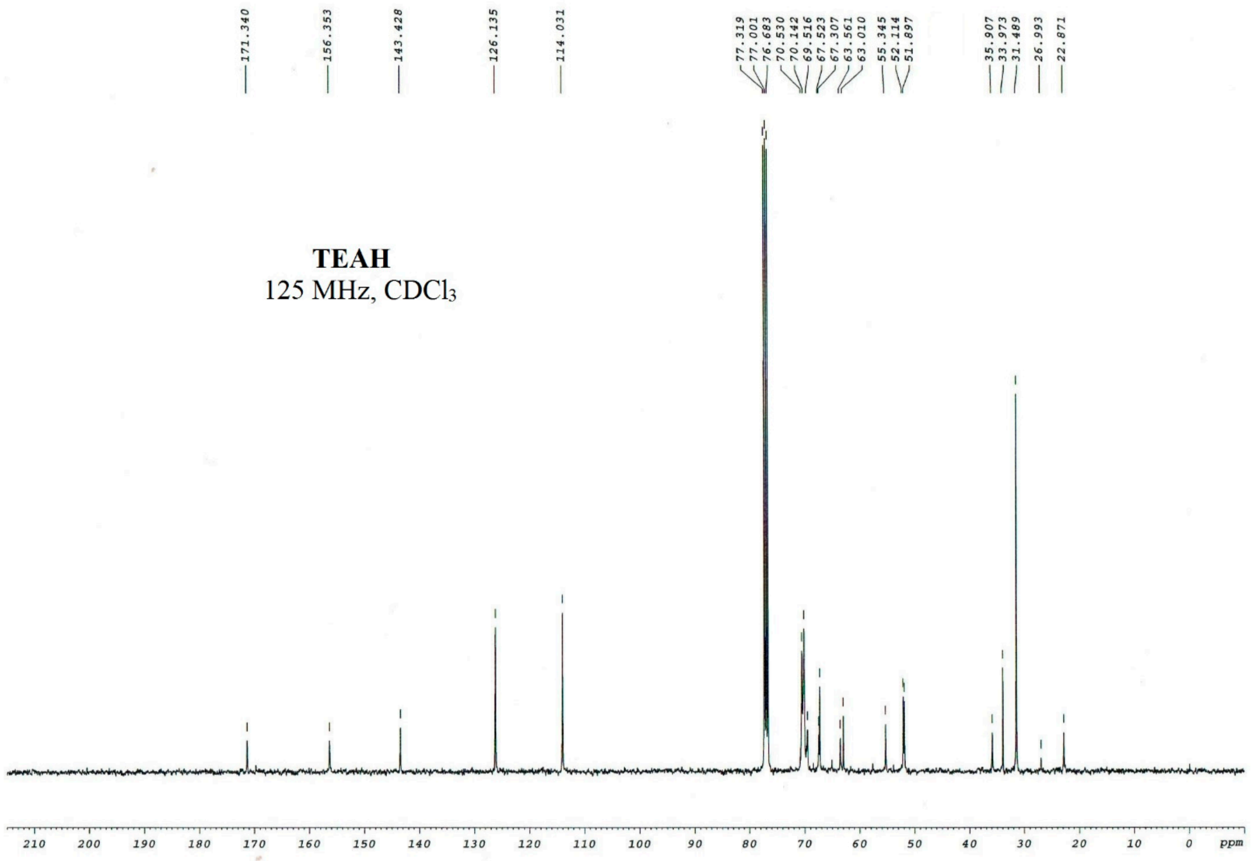

Figure S7. ^{13}C-NMR of zwitterionic surfactant (TEAH).

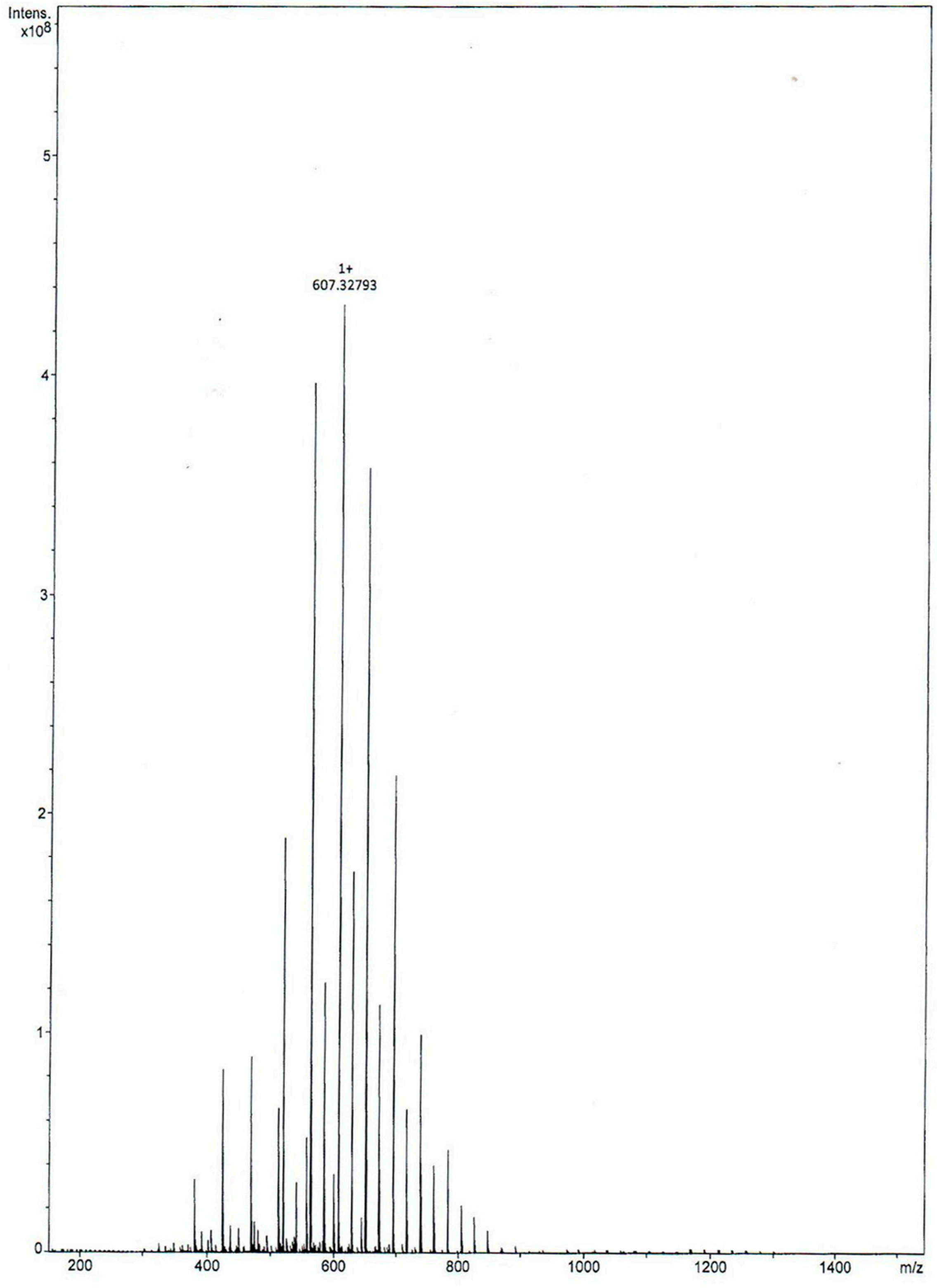

Figure S8. MALDI-TOF-MS spectra of zwitterionic surfactant (TEAH).

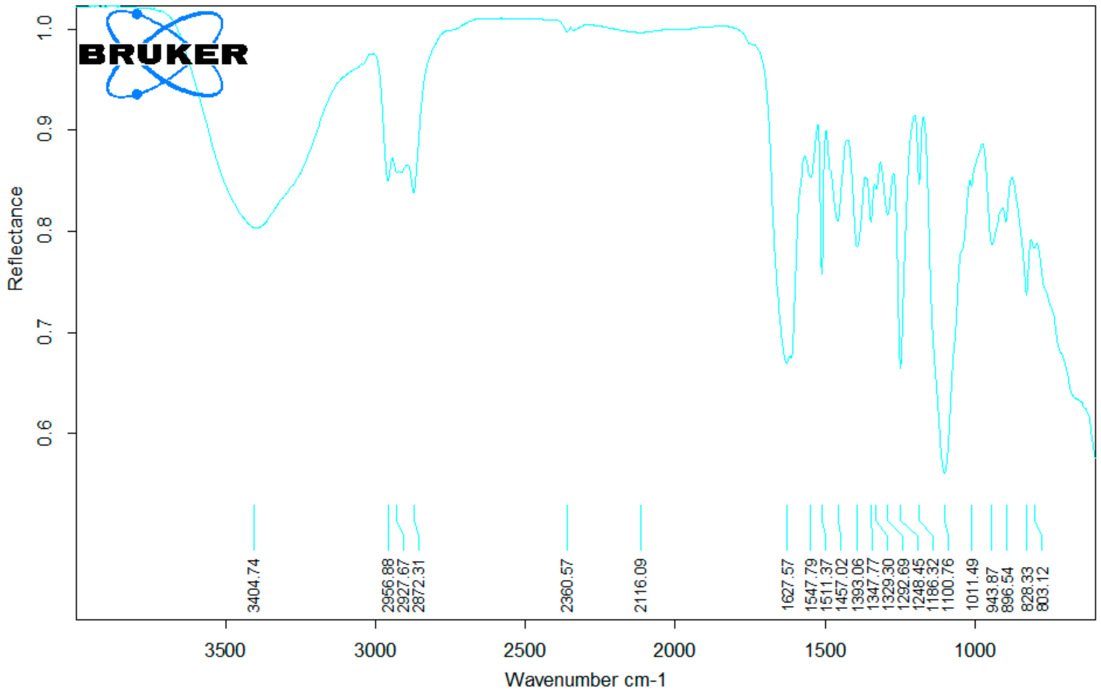

Figure S9. FT-IR spectrum of zwitterionic surfactants (NEAC).

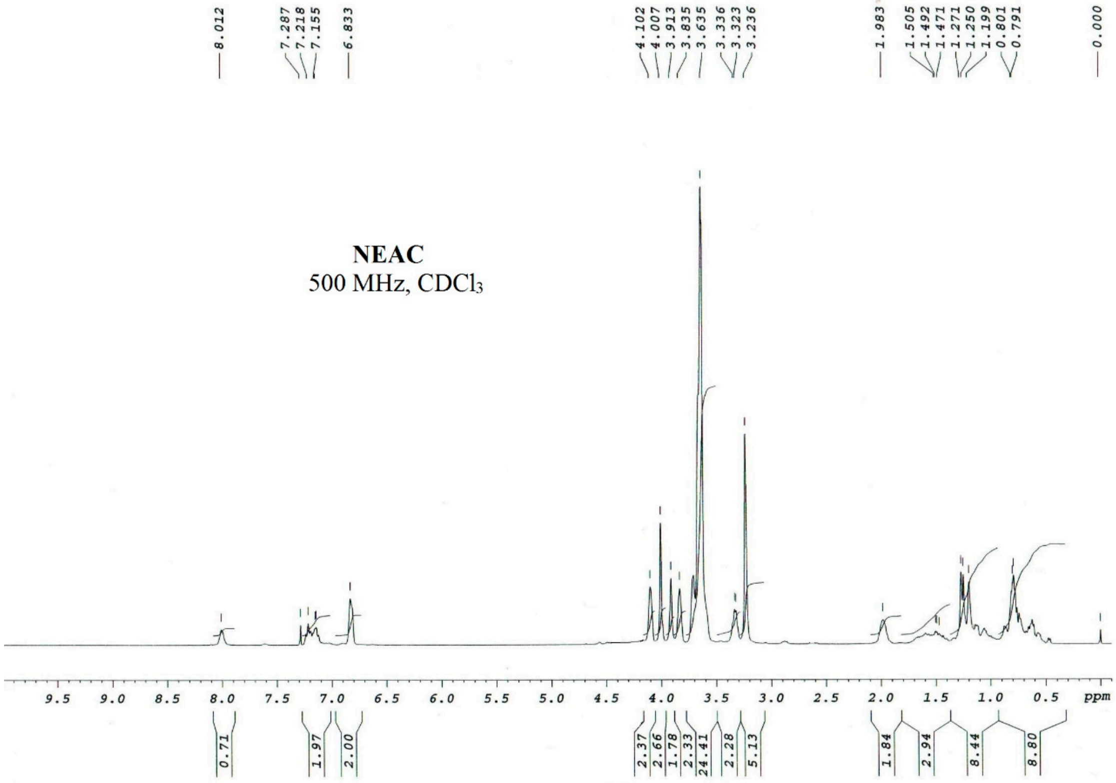

Figure S10. ^{1}H-NMR of zwitterionic surfactant (NEAC).

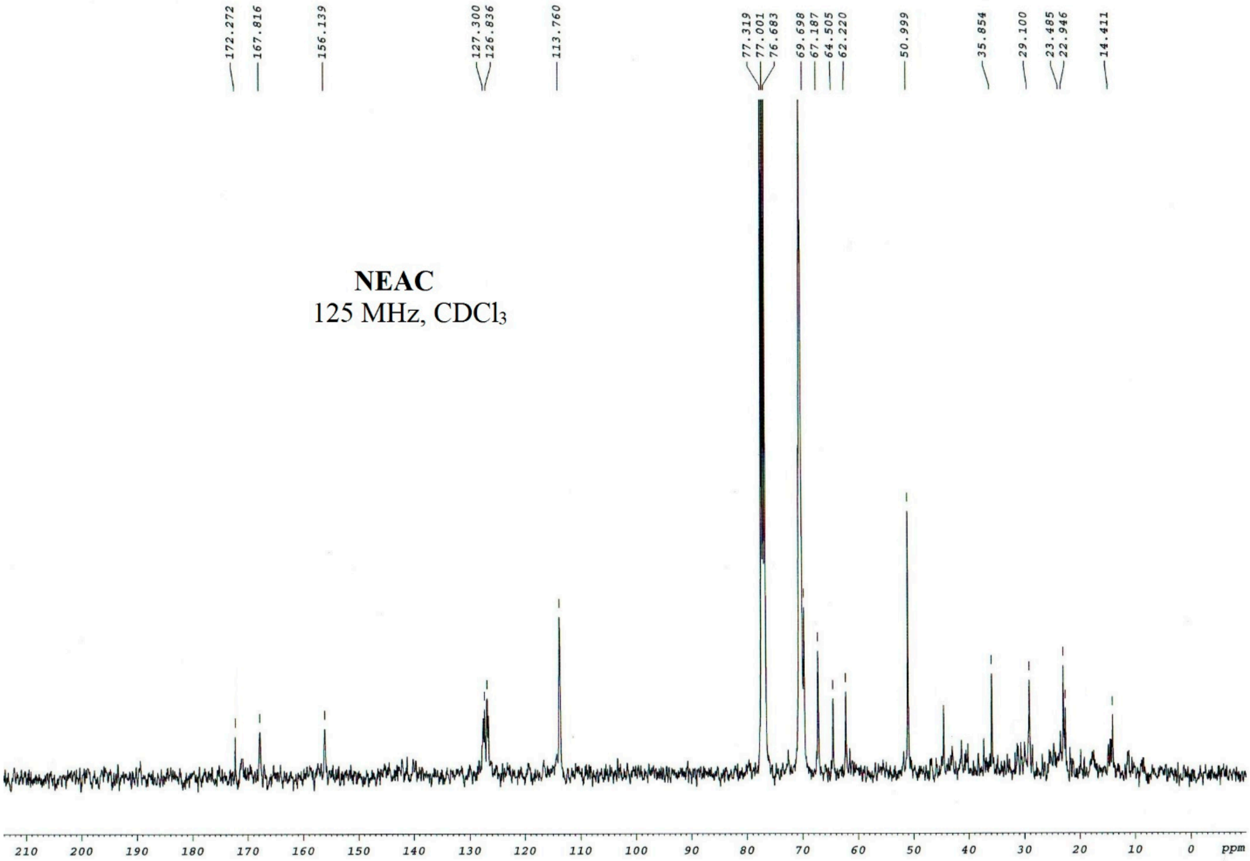

Figure S11. ^{13}C-NMR of zwitterionic surfactant (NEAC).

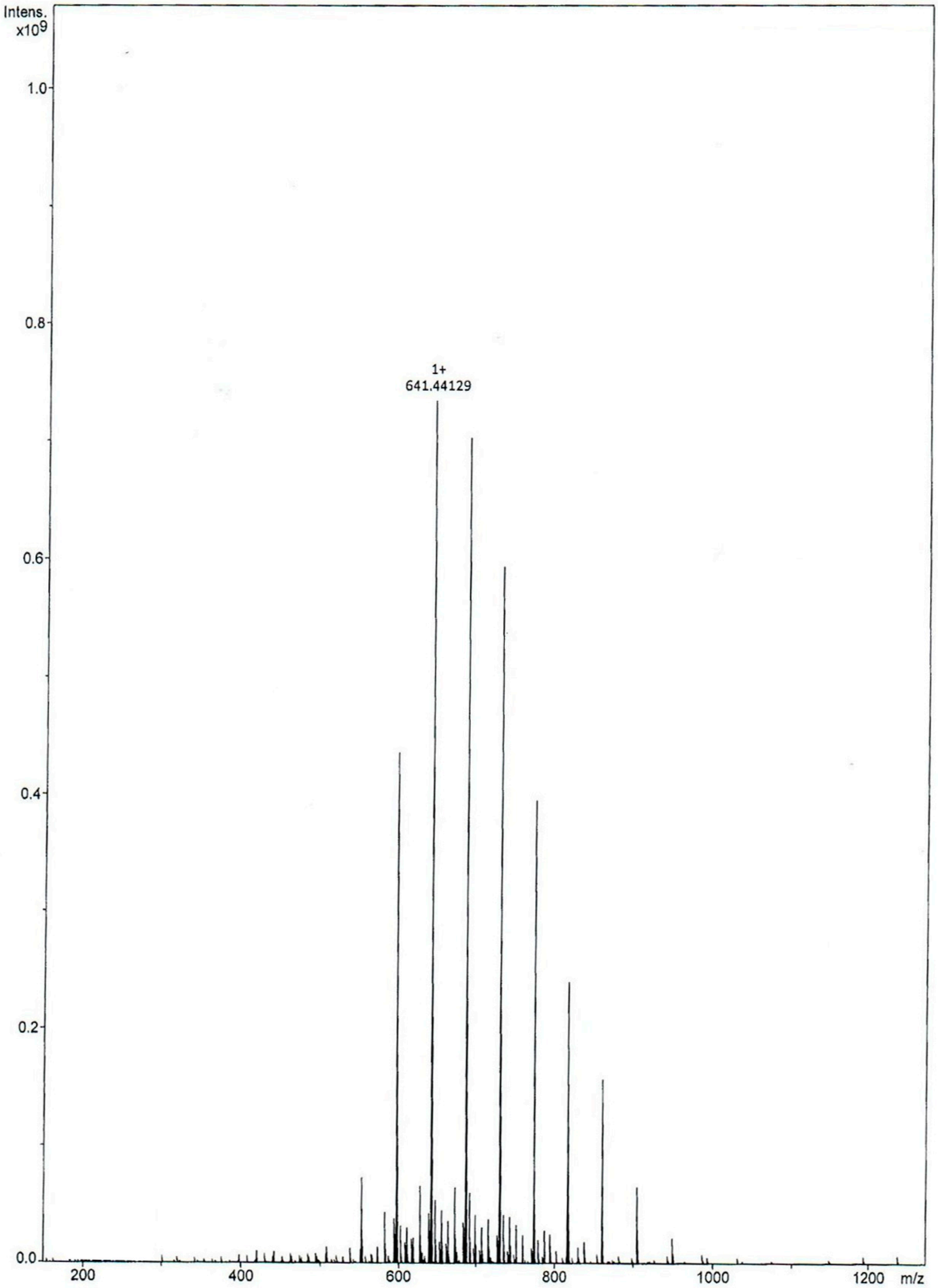

Figure S12. MALDI-TOF-MS spectra of zwitterionic surfactant (NEAC).

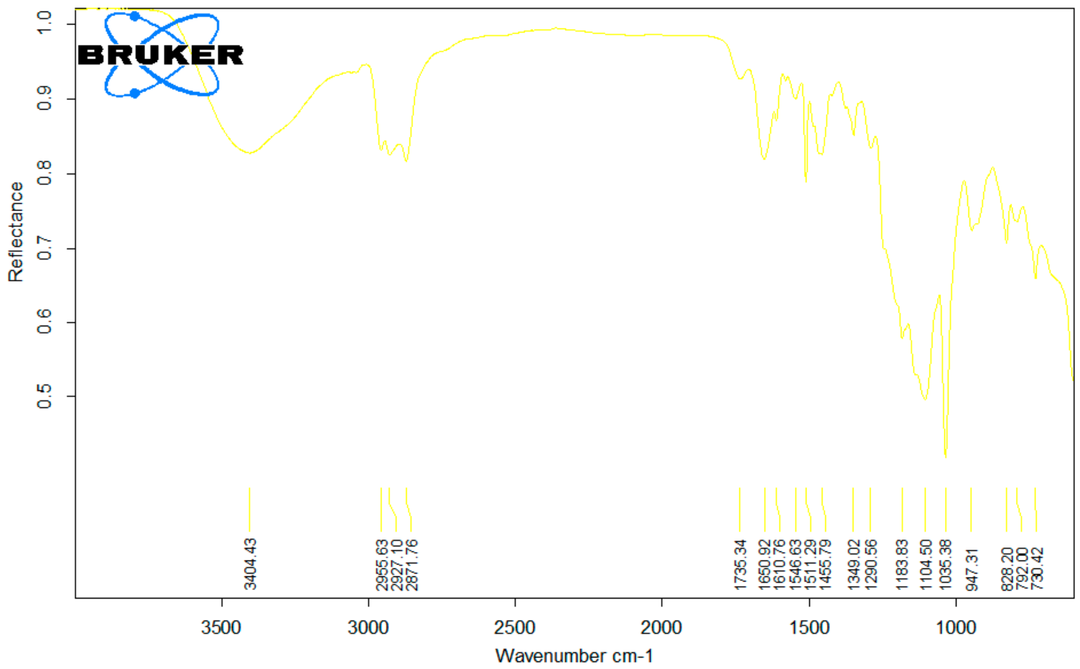

Figure S13. FT-IR spectrum of zwitterionic surfactants (NEAS).

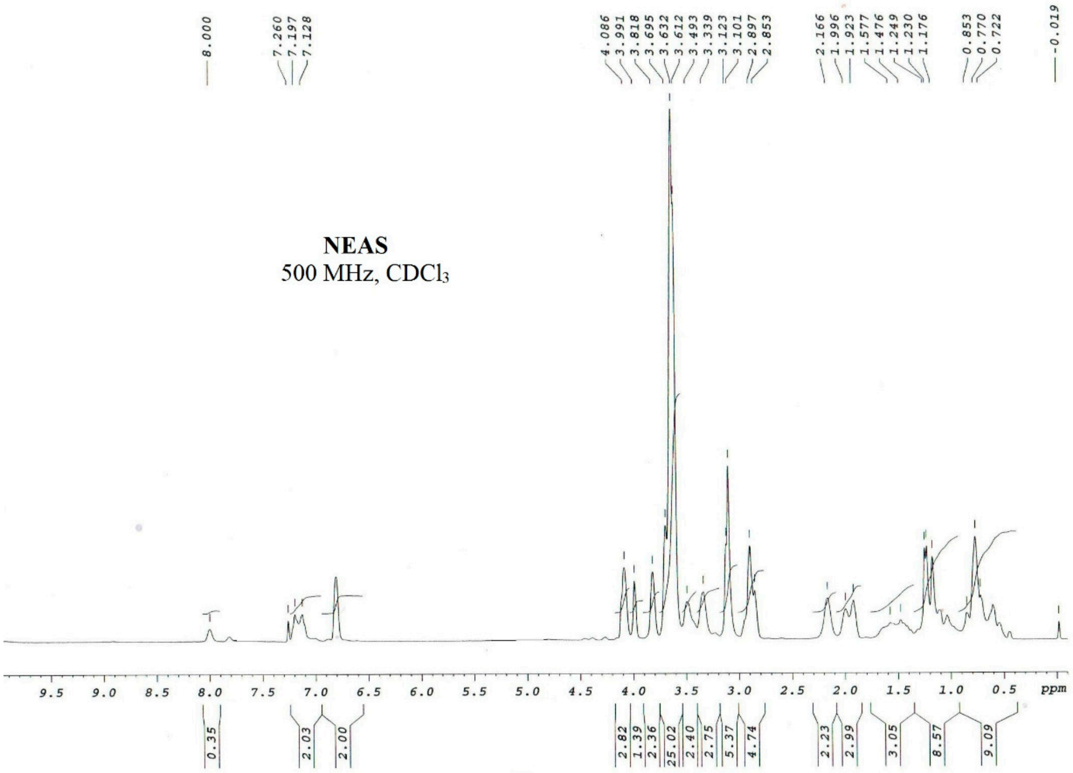

Figure S14. ^{1}H-NMR of zwitterionic surfactant (NEAS).

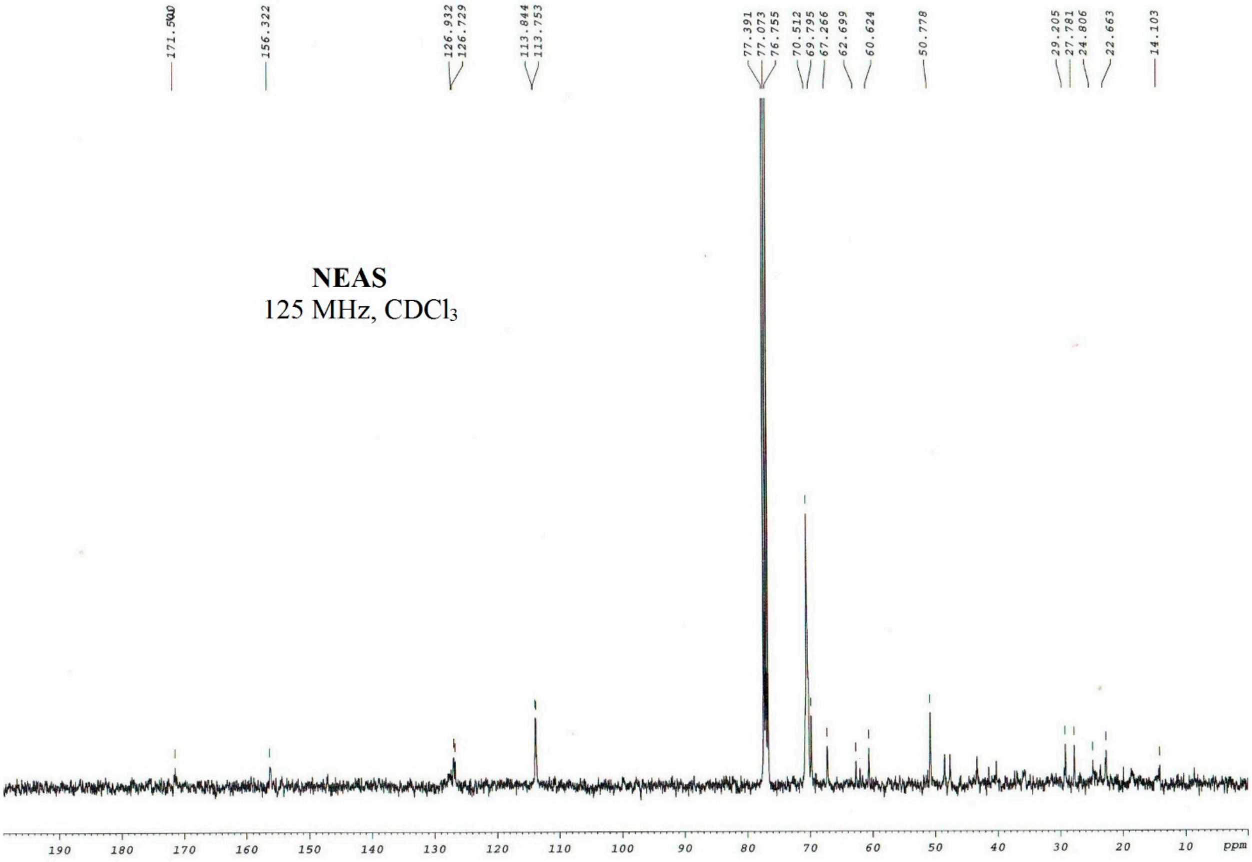

Figure S15. ^{13}C-NMR of zwitterionic surfactant (NEAS).

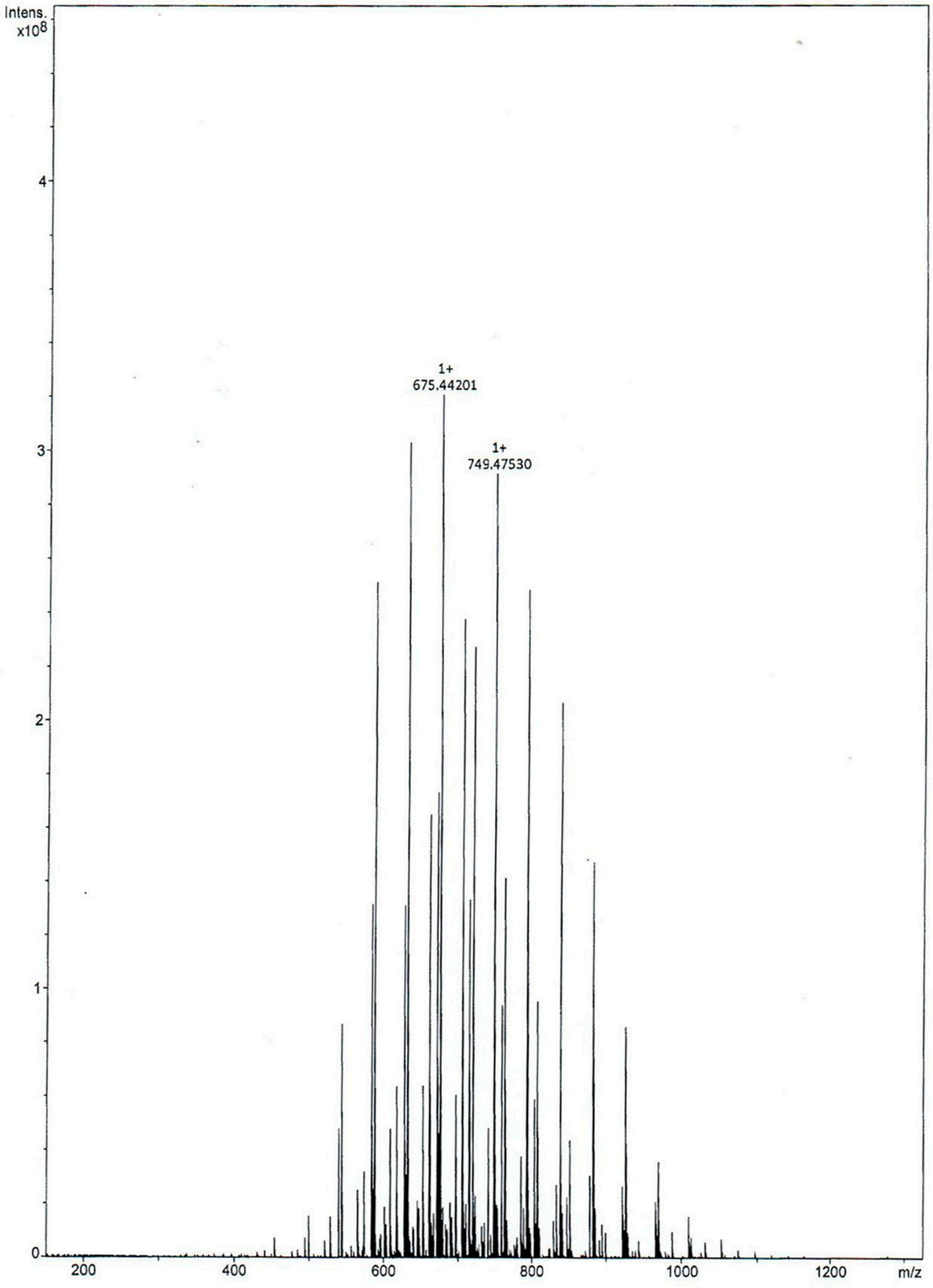

Figure S16. MALDI-TOF-MS spectra of zwitterionic surfactant (NEAS).

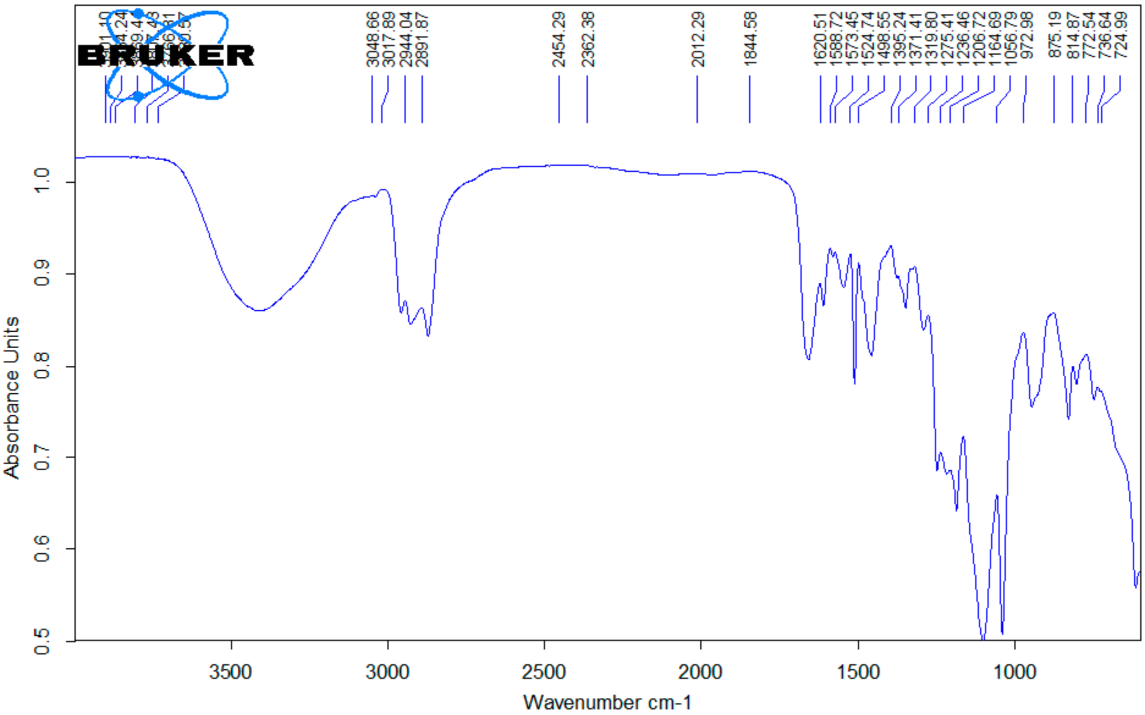

Figure S17. FT-IR spectrum of zwitterionic surfactants (NEAH).

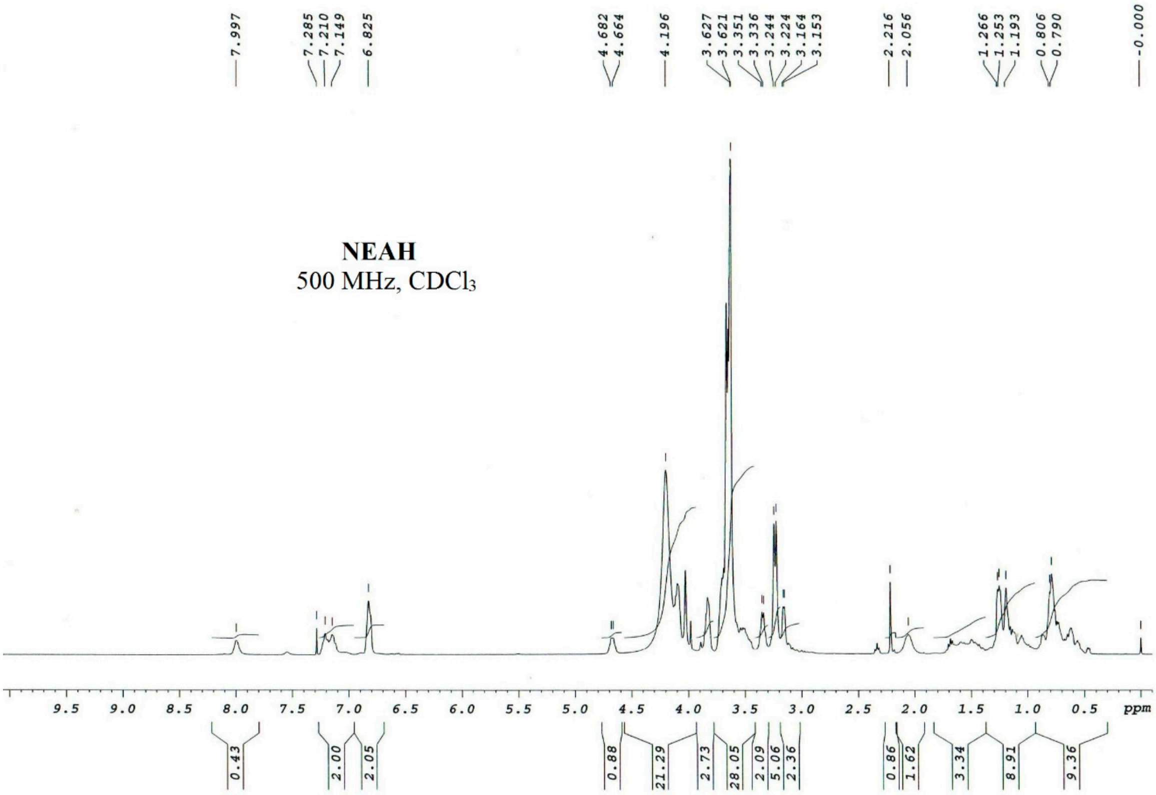

Figure S18. ^{1}H-NMR of zwitterionic surfactant (NEAH).

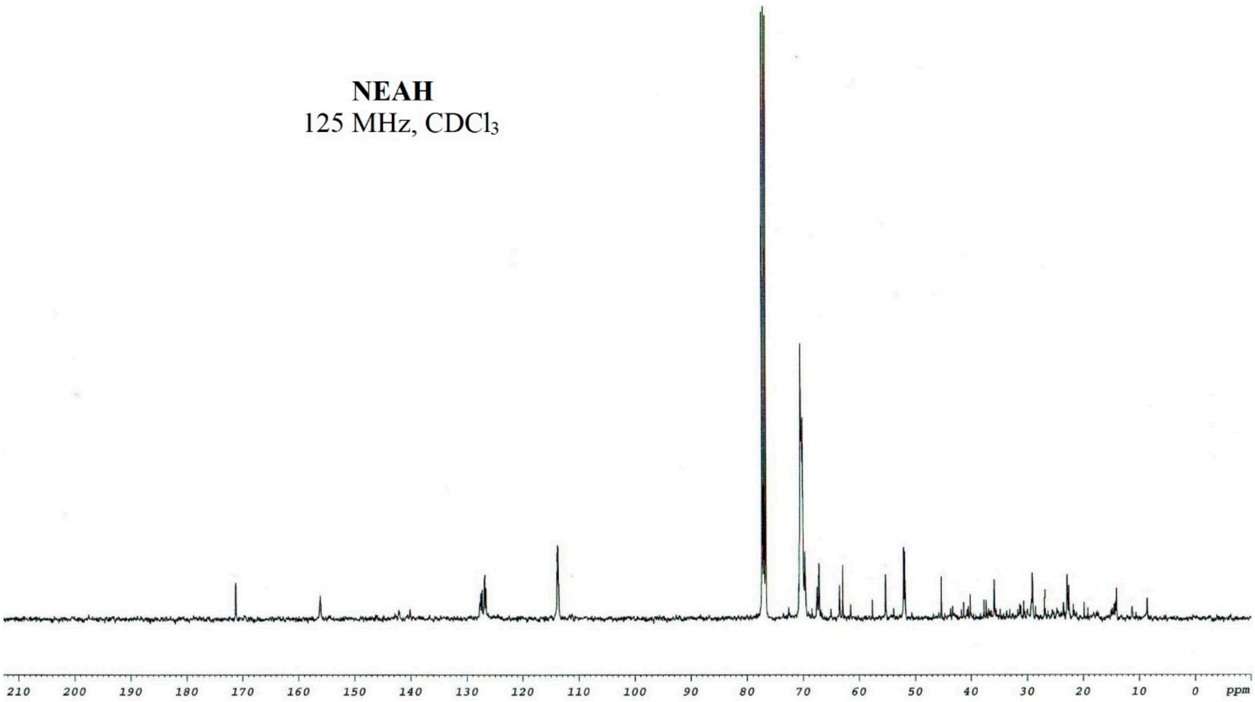

Figure S19. ^{13}C-NMR of zwitterionic surfactant (NEAH).

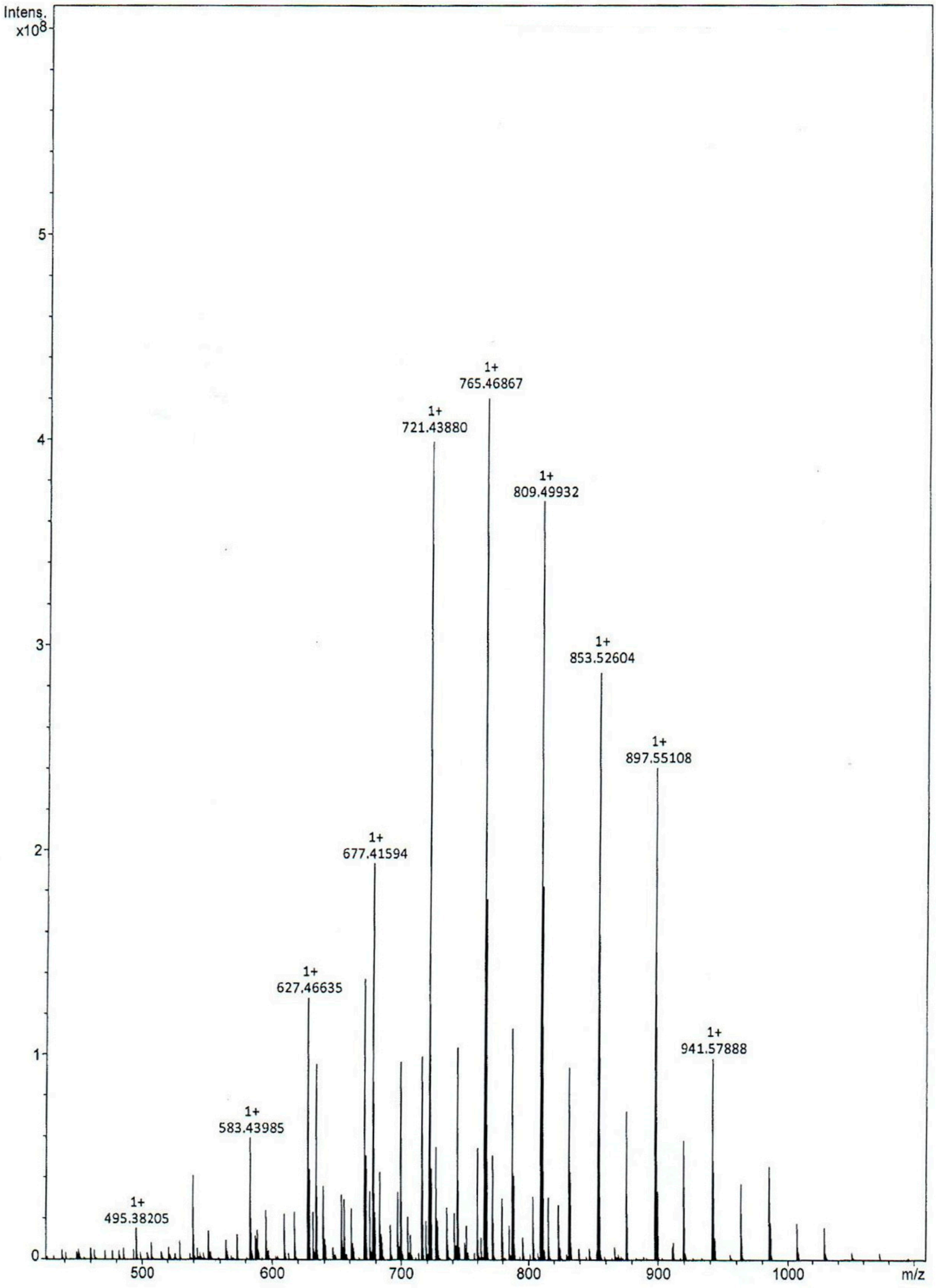

Figure S20. MALDI-TOF-MS spectra of zwitterionic surfactant (NEAH).
